# Supplementary material for: Qualitative and Quantitative Analysis of Ejiao-Related Animal Gelatins through Peptide Markers Using LC-QTOF-MS/MS and Scheduled Multiple Reaction Monitoring (MRM) by LC-QQQ-MS/MS
Source: Molecules. 2022 Jul 21;27(14):4643. doi: 10.3390/molecules27144643 (PMC9318382; doi:10.3390/molecules27144643)
Supplement: Supplementary file 1 [file molecules-27-04643-s001.zip › molecules-1645388-supplementary.pdf]

molecules

MDPI

Supplementary Materials

Qualitative and Quantitative Analysis of Ejiao-Related Animal Gelatins through Peptide Markers Using LC-QTOF-MS/MS and Scheduled Multiple Reaction Monitoring (MRM) by LC-QQQ-MS/MS

Wen-Jie Wu [1,†], Li-Feng Li [1,2,†], Hau-Yee Fung [1], Hui-Yuan Cheng [1], Hau-Yee Kong [1], Tin-Long Wong [1], Quan-Wei Zhang [1], Man Liu [1], Wan-Rong Bao [1], Chu-Ying Huo [1], Shang-Wei Guo [3], Hai-Bin Liu [3], Xiang-Shan Zhou [3,4,*], Deng-Feng Gao [2], Quan-Bin Han [1,2,*]

[1] School of Chinese Medicine, Hong Kong Baptist University, 7 Baptist University Road, Kowloon Tong, Hong Kong, 999077 China; 18482767@life.hkbu.edu.hk (W.-J.W.); 16483294@life.hkbu.edu.hk (L.-F.L.); 11018860@life.hkbu.edu.hk (H.-Y.F.); hycheng10@163.com (H.-Y.C.); 16223551@life.hkbu.edu.hk (H.-Y.K.); 15485021@life.hkbu.edu.hk (T.-L.W.); 18482422@life.hkbu.edu.hk (Q.-W.Z.); liuman@hkbu.edu.hk (M.L.); 16483502@life.hkbu.edu.hk (W.-R.B.); 20481969@life.hkbu.edu.hk (C.-Y.H.)

[2] Hong Kong Authentication Centre of Valuable Chinese Medicines, Hong Kong, 999077 China; gaodf@dongeejiao.com

[3] Shandong Technology Innovation Center of Gelatin-Based Traditional Chinese Medicine, Dong-E-E-Jiao Co., Ltd., No. 78, E-Jiao Street, Done-E Country, Shandong, 252200 China; guosw@dongee-jiao.com (S.-W.G.);
liuhaibin@dongeejiao.com (H.-B.L.)

[4] China Resources Biopharmaceutical Co., Ltd., Beijing, 100000 China

* Correspondence: zhouxiangshan1@crbiopharm.com (X.-S.Z.); simonhan@hkbu.edu.hk (Q.-B.H.); Tel.: +852-34112906 (Q.-B.H.); +86 10 5798 5166 (X.-S.Z.);
Fax: +852-34112461(Q.-B.H.)

† These authors contributed equally to this work.

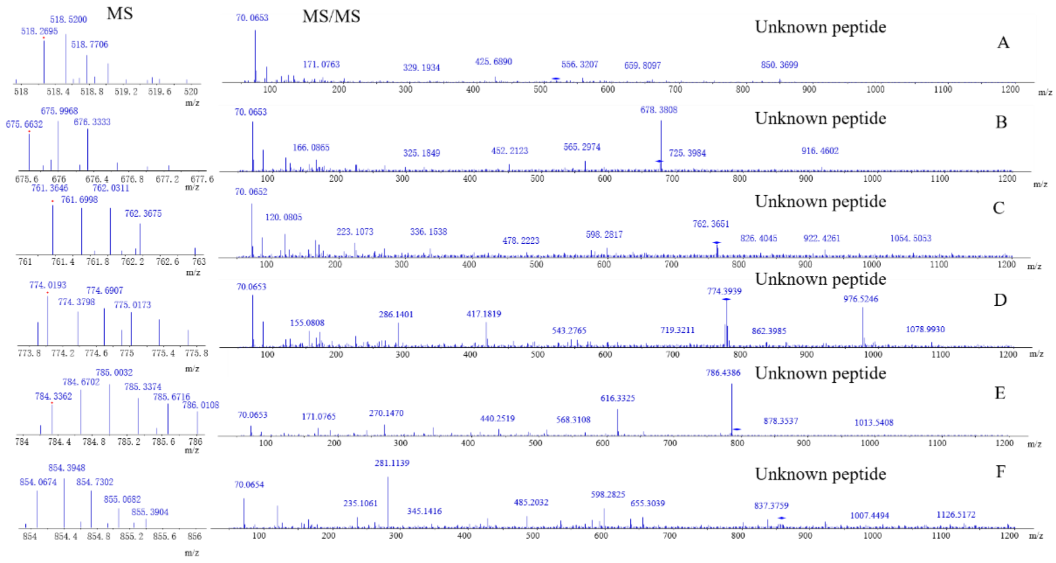

Figure S1. MS and MS/MS spectra of (A) donkey-specific marker 1, (B) donkey-specific marker 2 and (C) donkey-specific marker 3, (D) donkey-specific marker 5, (E) donkey-specific marker 6 and (F) donkey-specific marker 7.

Notes: A: (DM1) donkey-specific marker 1, m/z 518.2695±0.1, 18.92 min; B: (DM2) donkey-specific marker 2, m/z 675.6632±0.1, 23.36 min; C: (DM3) donkey-specific marker 3, m/z 761.3672±0.1, 24.24 min; D: (DM5) donkey-specific marker 5, m/z 774.0218±0.1, 23.36 min; E: (DM6) donkey-specific

marker 6, m/z 784.3392±0.1, 23.36 min; F: (DM7) donkey-specific marker 7, m/z 854.0603±0.1, 27.08 min. Three donkey-specific markers, including DM1 (KCSLDYGKDHEPVQVGPR), DM2 (CFWKYNGLPGSAFCFDK) and DM3 (NPTWNKPKPAYGHAGVGSMK), are determined by de novo MS/MS sequencing.

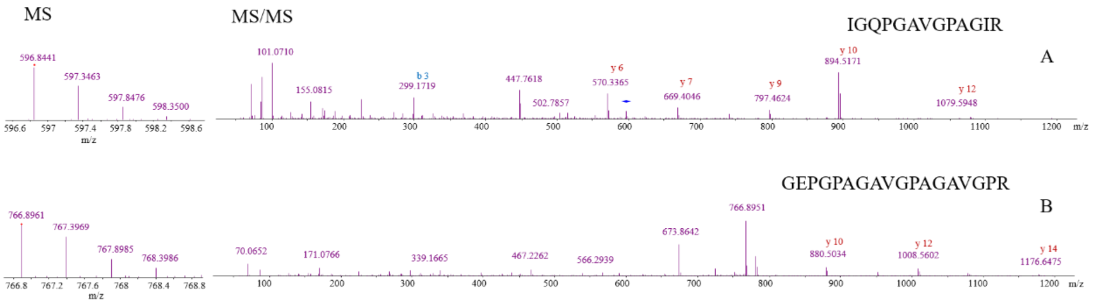

Figure S2. MS and MS/MS spectra of (A) cattle-specific marker 1 and (B) cattle-specific marker 3.

Notes: A: (CM1) cattle-specific marker 1, m/z 596.8454±0.1, 18.90 min; B: (CM3) cattle-specific marker 3, m/z 766.8957±0.1, 18.34 min.

Peptide sequences of A and B are obtained by searching protein databases. By comparing with Skyline software, their matched b and y ions are indicated.

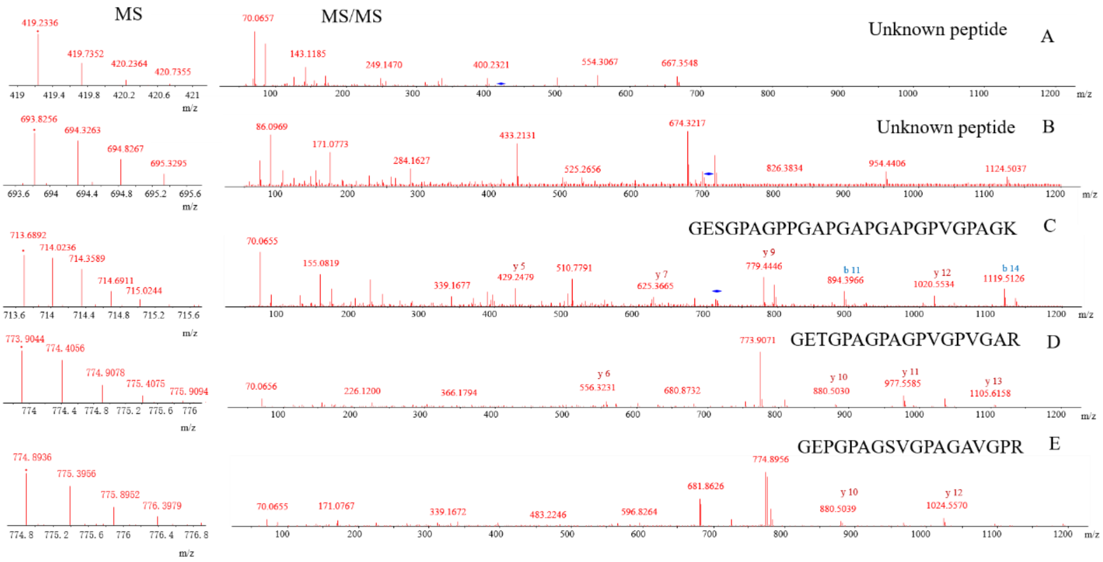

Figure S3. MS and MS/MS spectra of (A) pig-specific marker 1, (B) pig-specific marker 3, (C) pig-specific marker 4, (D) pig-specific marker 5 and (E) pig-specific marker 6.

Notes: A: (PM1) pig-specific marker 1, m/z 419.2446±0.1, 14.04 min; B: (PM3) pig-specific marker 3, m/z 693.8432±0.1, 26.7 min; C: (PM4) pig-specific marker 4, m/z 713.6902±0.1, 17.79 min; D: (PM5) pig-specific marker 5, m/z 773.9237±0.1, 18.51 min; E: (PM6) pig-specific marker 6, m/z 774.9121±0.2, 17.79 min.

Peptide sequences of C, D and E are obtained by searching protein databases. By comparing with Skyline software, their matched b and y ions are indicated. Another two pig-specific markers,

including PM1 (KLGPAGPR) and PM3 (GYGLAGLLGMGAHK), are determined by de novo MS/MS sequencing.

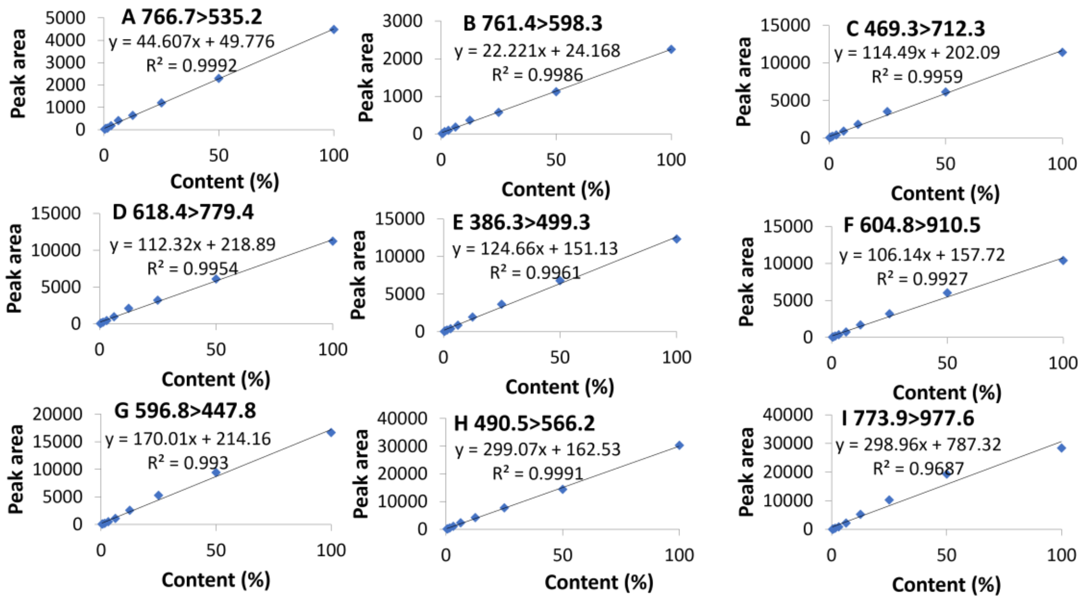

Figure S4. Linearity relationship between standard material content and specific-peptide marker including (A) donkey-specific marker 4, 766.7>535.2; (B) donkey-specific marker 3, 761.4>598.3; (C) donkey marker in the Chinese Pharmacopoeia, 469.3>712.3; (D) donkey marker in the Chinese Pharmacopoeia, 618.4>779.4; (E) horse-specific marker 1, 386.3>499.3; (F) cattle-specific marker 2, 604.8>910.5; (G) cattle-specific marker 1, 596.8>447.8; (H) pig-specific marker 2, 490.5>566.2; and (I) pig-specific marker 5, 773.9>977.6.

Notes: The ion pairs are detected by UPLC-QQQ-MS/MS in MRM mode.

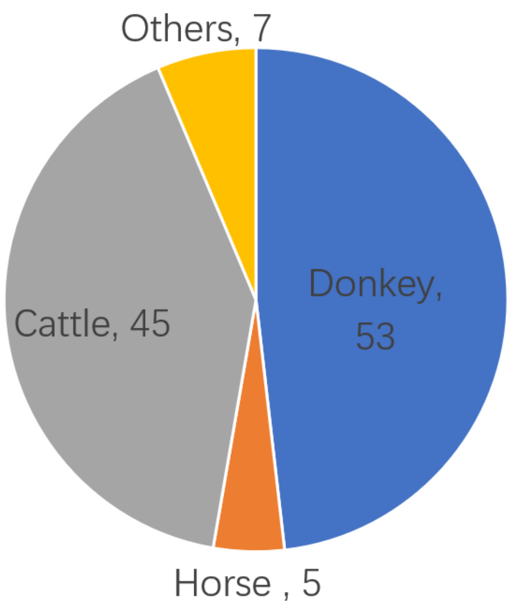

Figure S5. The authentication results of 110 commercial Ejiao samples.

Notes: Others includes 3 pig/horse-gelatin, 2 donkey/horse-gelatin, 1 cattle/horse- gelatin and 1 fake product which did not contain any gelatins.

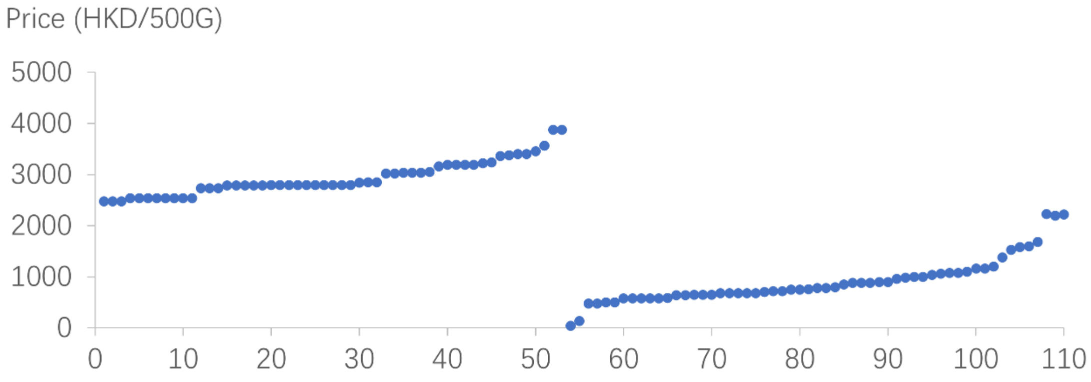

Figure S6. The relationships between authenticity and price.
Notes: 53 samples on the upper left are authentic products, and 57 samples on the lower right are fake products.

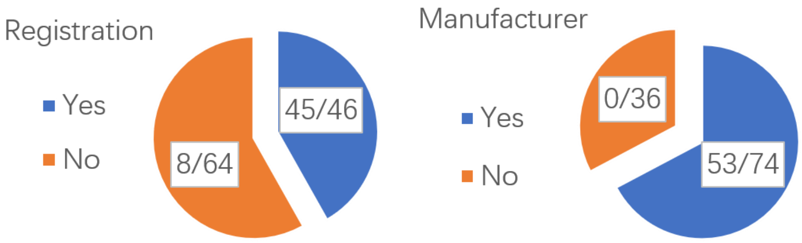

Figure S7. The relationships between authenticity and registration/manufacturers.
Notes: Registration represents having National Medical Products Administration (NMPA) approval number.

Supplementary Table S1

Markers selection for donkey-hide gelatin (Ejiao) from peptide fragments based on trypsin digestion followed by LC-Q-TOF-MS analysis.

Fig. 1 No.	Donkey marker No.	m/z	RT (min)	Charge	Donkey	Horse	Cattle	Pig
1	/	189.1288	5.31	1	✚	✚	✚	✚
2	/	228.6384	5.31	2	✚	✚	✚	✚
3	/	237.6447	5.31	2	✚	✚	✚	✚
4	/	233.1565	5.55	1	✚	✚	✚	✚
5	/	281.1566	5.59	1	✚	✚	✚	✚
6	/	260.2043	6.27	1	✚	✚	✚	✚
7	/	267.6266	6.27	2	✚	✚	✚	✚
8	/	288.2116	6.30		✚	✚	✚	✚
9	/	290.1784	6.30		✚	✚	✚	✚
10	/	276.1632	7.57	1	✚	✚	✚	✚
11	/	231.1771	7.77		✚	✚	✚	✚
12	/	216.6198	8.26	2	✚	✚	✚	✚
13	/	223.1141	8.27	1	✚	✚	✚	✚
14	/	236.6544	9.34	2	✚	✚	✚	✚
15	/	272.1795	9.40	1	✚	✚	✚	✚
16	/	260.1678	9.58	1	✚	✚	✚	✚
17	/	277.1257	9.74	1	✚	✚	✚	✚
18	/	237.6260	10.08	2	✚	✚	✚	✚
19	/	242.1565	10.08	1	✚	✚	✚	✚
20	/	249.6631	10.08	2	✚	✚	✚	✚
21	/	270.2001	10.08	1	✚	✚	✚	✚
22	/	274.1954	10.08	1	✚	✚	✚	✚
23	/	281.1213	10.08	1	✚	✚	✚	✚
24	/	295.1740	10.08	1	✚	✚	✚	✚
25	/	258.1766	10.10	2	✚	✚	✚	✚
26	/	231.1762	10.44	1	✚	✚	✚	✚
27	/	219.1404	10.98	1	✚	✚	✚	✚
28	/	295.1730	10.98	1	✚	✚	✚	✚
29	/	229.6280	12.09	2	✚	✚	✚	✚
30	/	272.6535	12.27	2	✚	✚	✚	✚
31	/	283.8095	12.27	2	✚	✚	✚	✚
32	/	292.6630	12.27	2	✚	✚	✚	✚
33	/	278.6662	12.50	2	✚	✚	✚	✚
34	/	297.6402	12.50	2	✚	✚	✚	✚

35	/	265.1621	13.32	1	✚	✚	✚	✚
36	/	245.1925	14.24	1	✚	✚	✚	✚
37	/	245.1925	15.38	1	✚	✚	✚	✚
38	/	243.1408	15.75	1	✚	✚	✚	✚
39	/	279.1418	17.53	1	✚	✚	✚	✚
40	/	279.1777	18.20	1	✚	✚	✚	✚
41	/	288.2009	20.49		✚	✚	✚	✚
42	/	234.1024	22.27		✚	✚	✚	✚
43	/	224.1352	31.00	1	✚	✚	✚	✚
44	/	250.1855	32.78	1	✚	✚	✚	✚
45	/	329.2007	5.31	1	✚	✚	✚	✚
46	/	345.2333	5.31	1	✚	✚	✚	✚
47	/	391.1910	5.31	1	✚	✚	✚	✚
48	/	321.1817	5.59	1	✚	✚	✚	✚
49	/	324.1811	5.59	2	✚	✚	✚	✚
50	/	372.6956	5.59	2	✚	✚	✚	✚
51	/	301.1832	6.30	2	✚	✚	✚	✚
52	/	303.6207	6.30	1	✚	✚	✚	✚
53	/	306.1739	6.30		✚	✚	✚	✚
54	/	317.1910	6.30	3	✚	✚	✚	✚
55	/	323.1587	6.30		✚	✚	✚	✚
56	/	323.4940	6.30		✚	✚	✚	✚
57	/	329.1909	6.30	2	✚	✚	✚	✚
58	/	369.7033	6.30	2	✚	✚	✚	✚
59	/	373.2899	6.30	1	✚	✚	✚	✚
60	/	388.2293	6.30		✚	✚	✚	✚
61	/	398.1658	6.30		✚	✚	-	✚
62	/	347.2014	7.77	1	✚	✚	✚	✚
63	/	375.2342	7.77	1	✚	✚	✚	✚
64	/	325.6760	8.17	2	✚	✚	-	-
65	/	301.6758	8.27	2	✚	✚	✚	✚
66	/	320.1866	8.27	1	✚	✚	✚	-
67	/	365.8721	8.27		✚	✚	✚	-
68	/	385.2063	8.27	2	✚	✚	✚	✚
69	/	384.2149	8.64	2	✚	✚	✚	-
70	/	395.6893	8.94	2	✚	✚	✚	✚
71	/	385.2661	9.40	1	✚	✚	✚	✚
72	/	393.2091	9.40	2	✚	✚	✚	✚
73	/	345.1529	9.63	1	✚	✚	✚	✚
74	/	350.1806	9.63	1	✚	✚	✚	✚
75	/	322.6805	10.06	2	✚	✚	✚	✚
76	/	302.1801	10.08	1	✚	✚	✚	✚

77	/	359.2392	10.08	1	✚	✚	✚	✚
78	/	385.2297	10.08	1	✚	✚	✚	✚
79	/	398.2194	10.08		✚	✚	✚	-
80	/	372.2159	10.36	2	✚	✚	✚	✚
81	/	390.2222	10.60	2	✚	✚	✚	✚
82	/	339.1935	10.87	2	✚	✚	✚	✚
83	/	371.2385	10.98	1	✚	✚	✚	✚
84	/	379.7035	10.98	2	✚	✚	✚	✚
85	/	389.2488	11.01	1	✚	✚	✚	✚
86	/	393.2267	11.01	2	✚	✚	✚	-
87	/	314.7013	11.46	2	✚	✚	✚	-
88	/	347.7072	11.46	2	✚	✚	✚	✚
89	/	377.1953	11.46	1	✚	✚	✚	✚
90	/	305.6956	11.52	2	✚	✚	✚	✚
91	/	339.6729	11.52	2	✚	✚	✚	✚
92	/	314.6831	12.07	2	✚	✚	✚	✚
93	/	322.8205	12.27	3	✚	✚	✚	✚
94	/	327.8098	12.27	3	✚	✚	✚	✚
95	/	343.2051	12.27		✚	✚	✚	✚
96	/	388.2296	12.27		✚	✚	✚	✚
97	/	309.6738	12.35	2	✚	✚	✚	✚
98	/	374.7011	12.41	2	✚	✚	✚	✚
99	/	300.6175	12.78	2	✚	✚	✚	✚
100	/	320.2031	12.78	3	✚	✚	✚	✚
101	/	330.2011	12.78	2	✚	✚	✚	✚
102	/	390.8836	12.91	3	✚	✚	-	-
103	/	392.2315	13.05	2	✚	✚	✚	✚
104	/	321.1797	13.31	2	✚	✚	✚	-
105	/	369.2061	13.59	3	✚	✚	✚	✚
106	/	377.1926	13.59	1	✚	✚	✚	✚
107	/	381.8550	13.59	3	✚	✚	✚	✚
108	/	385.5505	13.59	3	✚	✚	-	-
109	/	386.8436	13.59	3	✚	✚	✚	✚
110	/	350.6348	14.08	2	✚	✚	✚	✚
111	/	359.6404	14.08	2	✚	✚	✚	✚
112	/	378.6137	14.08	2	✚	✚	✚	✚
113	/	398.2504	14.18	1	✚	✚	✚	✚
114	/	301.6768	14.24	2	✚	✚	✚	✚
115	/	332.2266	14.24	1	✚	✚	✚	✚
116	/	374.8536	14.46	3	✚	✚	✚	✚
117	/	379.8415	14.46	3	✚	✚	✚	✚
118	/	382.5030	14.46	3	✚	✚	✚	✚

119	/	396.2707	14.62	1	✚	✚	✚	✚
120	/	320.1733	14.88	1	✚	✚	✚	✚
121	/	353.4912	14.88	3	✚	✚	✚	✚
122	/	372.8516	14.88	3	✚	✚	-	-
123	/	377.8407	14.88	3	✚	✚	-	-
124	/	329.2282	15.00	2	✚	✚	✚	✚
125	/	383.7419	15.00	2	✚	✚	✚	-
126	/	398.2272	15.00	3	✚	✚	✚	✚
127	/	371.7236	16.41	2	✚	✚	✚	✚
128	/	312.8329	16.42	3	✚	✚	✚	✚
129	/	399.2711	17.34	1	✚	✚	✚	✚
130	/	366.2176	17.35	2	✚	✚	✚	✚
131	/	302.2166	18.21	1	✚	✚	✚	✚
132	/	391.2092	18.32	1	✚	✚	✚	✚
133	/	332.2273	18.89	1	✚	✚	✚	✚
134	/	374.2389	18.89	1	✚	✚	✚	✚
135	/	302.2159	19.22	1	✚	✚	✚	✚
136	/	316.2312	19.63	1	✚	✚	✚	✚
137	/	387.8462	19.63	3	✚	✚	✚	✚
138	/	330.1635	19.95	1	✚	✚	✚	✚
139	/	337.1392	20.13	2	✚	✚	-	-
140	/	392.9530	20.13	4	✚	✚	-	-
141	/	396.6962	20.13	4	✚	✚	✚	-
142	/	344.2632	20.16	1	✚	✚	✚	✚
143	/	338.8360	21.02		✚	✚	-	-
144	/	335.1611	21.76	3	✚	✚	✚	✚
145	/	306.1484	23.08	2	✚	✚	✚	✚
146	/	393.7321	23.66	2	✚	✚	-	-
147	/	379.2181	24.94	2	✚	✚	✚	✚
148	/	422.2127	5.31	1	✚	✚	✚	✚
149	/	474.2799	5.31	1	✚	✚	✚	✚
150	/	459.2685	5.59	1	✚	✚	✚	✚
151	/	473.2346	5.59		✚	✚	✚	-
152	/	490.2600	5.59		✚	✚	✚	✚
153	/	431.2478	6.30	1	✚	✚	✚	✚
154	/	443.2353	6.30	1	✚	✚	✚	✚
155	/	447.2105	6.30	1	✚	✚	✚	✚
156	/	462.2302	6.30	1	✚	✚	✚	✚
157	/	484.2314	6.30	2	✚	✚	✚	✚
158	/	445.2527	7.80	1	✚	✚	✚	✚
159	/	499.2625	7.80	1	✚	✚	✚	✚
160	/	432.2315	8.27	1	✚	✚	✚	✚

161	/	446.2717	8.27	1	+	+	+	+
162	/	426.7299	8.94	2	+	+	+	+
163	/	456.2434	8.94	2	+	+	+	+
164	/	400.2297	9.40	1	+	+	+	+
165	/	404.2236	9.40	1	+	+	+	+
166	/	472.2992	9.40	1	+	+	+	+
167	/	405.2186	9.63	2	+	+	+	+
168	/	421.7331	9.63	2	+	+	+	+
169	/	498.7538	9.63	2	+	+	+	+
170	/	402.2567	10.08	1	+	+	+	+
171	/	444.2303	10.08	2	+	+	+	+
172	/	469.2535	10.08	2	+	+	-	-
173	/	474.2429	10.08	1	+	+	+	+
174	/	405.2085	10.36		+	+	-	-
175	/	418.7339	10.36	2	+	+	+	+
176	/	448.2467	10.36	2	+	+	+	+
177	/	475.7502	10.57	2	+	+	+	+
178	/	434.7471	10.81	2	+	+	+	+
179	/	411.2322	10.98	1	+	+	+	+
180	/	427.7393	10.98	2	+	+	-	-
181	/	459.2678	10.98	1	+	+	+	+
182	/	427.7383	11.01	2	+	+	-	-
183	/	467.7517	11.01	2	+	+	+	+
184	/	461.2554	11.46	2	+	+	-	-
185	/	446.7472	11.52	2	+	+	+	+
186	/	468.2369	11.52	1	+	+	+	+
187	/	474.2428	11.52	1	+	+	+	+
188	/	458.2476	12.10	2	+	+	+	+
189	/	492.5855	12.10	3	+	+	-	-
190	/	406.2358	12.21	2	+	+	+	+
191	/	464.7522	12.26	2	+	+	+	+
192	/	414.2457	12.27	1	+	+	+	+
193	/	442.7441	12.27	2	+	+	+	+
194	/	453.2203	12.27		+	+	+	+
195	/	457.7509	12.27	2	+	+	+	+
196	/	459.1892	12.27	2	+	+	+	+
197	/	474.2679	12.27	2	+	+	+	+
198	/	474.2682	12.27	1	+	+	+	+
199	/	476.2476	12.35	2	+	+	+	+
200	/	421.2412	12.45	2	+	+	+	+
201	/	400.2298	12.78	2	+	+	+	+
202	/	430.2411	12.78	1	+	+	+	+

203	/	479.7979	12.78	2	✚	✚	✚	✚
204	/	471.7388	12.84	2	✚	✚	✚	✚
205	/	420.1979	13.01	1	✚	✚	✚	✚
206	/	469.2711	13.03	2	✚	✚	-	-
207	/	456.2456	13.15	2	✚	✚	✚	✚
208	/	406.2352	13.25	2	✚	✚	✚	-
209	/	472.2880	13.31	1	✚	✚	✚	✚
210	/	431.2253	13.59	2	✚	✚	✚	✚
211	/	447.2085	13.59	1	✚	✚	✚	✚
212	/	478.7565	13.59	2	✚	✚	✚	✚
213	/	454.7644	13.80	2	✚	✚	✚	✚
214	/	464.2263	13.90	1	✚	✚	✚	✚
215	/	413.2410	14.18	2	✚	✚	✚	✚
216	/	499.2998	14.24	1	✚	✚	-	-
217	/	414.2108	14.52	2	✚	✚	-	-
218	/	447.2542	14.60	1	✚	✚	✚	✚
219	/	486.3042	14.60	1	✚	✚	✚	✚
220	/	446.7659	14.62	2	✚	✚	✚	✚
221	/	486.3052	14.62	1	✚	✚	✚	✚
222	/	414.2413	14.88	3	✚	✚	✚	✚
223	/	457.2030	14.88	2	✚	✚	✚	✚
224	/	465.2464	14.88	1	✚	✚	✚	✚
225	/	403.2657	15.00	1	✚	✚	✚	✚
226	/	410.8747	15.00	3	✚	✚	✚	✚
227	/	415.8626	15.00	3	✚	✚	✚	✚
228	/	421.2612	15.00	2	✚	✚	✚	✚
229	/	412.2540	15.75	2	✚	✚	✚	✚
230	/	407.2195	15.76	2	✚	✚	✚	✚
231	/	403.2657	16.04	1	✚	✚	-	-
232	/	412.2536	16.04	2	✚	✚	✚	✚
233	/	449.7704	16.48	2	✚	✚	✚	✚
234	/	478.2575	16.50	2	✚	✚	-	✚
235	/	446.2722	16.69	1	✚	✚	✚	✚
236	/	434.2619	17.34	2	✚	✚	✚	✚
237	/	479.2622	17.50	1	✚	✚	✚	✚
238	/	418.2195	17.95	1	✚	✚	✚	✚
239	/	467.7294	17.95	2	✚	✚	✚	✚
240	/	473.2079	18.21	2	✚	✚	✚	✚
241	/	441.7730	18.54	2	✚	✚	✚	✚
242	/	460.4769	18.89	4	✚	✚	-	-
243	/	464.4752	18.89	4	✚	✚	-	-
244	/	472.5596	18.89	3	✚	✚	✚	✚

245	/	476.2273	18.89	1	✚	✚	✚	✚
246	/	462.2472	19.19	1	✚	✚	✚	✚
247	/	493.2424	19.19	1	✚	✚	✚	✚
248	/	455.2042	19.61	1	✚	✚	✚	✚
249	/	404.4604	19.63	4	✚	✚	✚	✚
250	/	407.9573	19.63	4	✚	✚	✚	✚
251	/	411.6992	19.63	4	✚	✚	✚	✚
252	/	446.5774	19.63	3	✚	✚	-	-
253	/	459.2278	19.63	3	✚	✚	✚	✚
254	/	421.2432	20.13	2	✚	✚	✚	✚
255	/	441.2521	20.49	2	✚	✚	✚	✚
256	/	444.2936	20.49	1	✚	✚	✚	✚
257	/	430.2775	20.63	1	✚	✚	✚	✚
258	/	429.2826	21.01	1	✚	✚	✚	✚
259	/	499.7645	21.01	2	✚	✚	✚	✚
260	/	472.2893	21.02	1	✚	✚	✚	✚
261	/	488.7716	21.02	2	✚	✚	-	-
262	/	413.2866	21.76	1	✚	✚	✚	✚
263	/	480.2565	21.76	1	✚	✚	✚	✚
264	/	431.5276	21.95	3	✚	✚	✚	✚
265	/	459.2935	21.96	1	✚	✚	✚	✚
266	/	451.2280	22.27	1	✚	✚	✚	✚
267	/	429.2457	22.30	1	✚	✚	✚	✚
268	/	402.5164	22.63	3	✚	✚	✚	✚
269	/	415.2447	22.63	1	✚	✚	✚	✚
270	/	480.7737	22.63	2	✚	✚	-	-
271	/	491.7648	22.63	2	✚	✚	-	-
272	/	456.2925	23.26	1	✚	✚	✚	✚
273	/	437.7673	31.77	2	✚	✚	✚	✚
274	/	526.2750	5.31	1	✚	✚	✚	✚
275	/	573.2644	5.31	1	✚	✚	✚	✚
276	/	578.2752	5.31	1	✚	✚	✚	✚
277	/	558.3128	5.59		✚	✚	✚	✚
278	/	501.2429	6.30	1	✚	✚	✚	✚
279	/	534.2432	6.30	1	✚	✚	✚	✚
280	/	568.2868	6.30	1	✚	✚	✚	✚
281	/	584.3129	6.30		✚	✚	✚	✚
282	/	515.3429	10.06	1	✚	✚	✚	✚
283	/	569.7932	10.06	2	✚	✚	✚	✚
284	/	581.3193	11.52	1	✚	✚	✚	✚
285	/	527.2957	12.10	1	✚	✚	✚	✚
286	/	570.3125	12.10	1	✚	✚	✚	✚

287	/	583.8135	12.10	2	✚	✚	✚	✚
288	/	596.3298	12.10	1	✚	✚	✚	✚
289	/	531.2907	12.27	1	✚	✚	✚	✚
290	/	571.2819	12.27	1	✚	✚	✚	✚
291	/	578.3000	12.27	2	✚	✚	✚	✚
292	/	536.7798	12.35	2	✚	✚	✚	-
293	/	544.2973	12.40	1	✚	✚	✚	✚
294	/	589.2899	12.40	3	✚	✚	-	✚
295	/	504.7541	12.50	2	✚	✚	✚	-
296	/	578.3056	12.53	1	✚	✚	✚	✚
297	/	556.3212	12.55	1	✚	✚	✚	✚
298	/	552.6142	12.58	3	✚	✚	✚	✚
299	/	538.2274	12.65	2	✚	✚	✚	✚
300	/	531.7855	12.78	2	✚	✚	✚	✚
301	/	562.2794	12.78	1	✚	✚	✚	✚
302	/	531.7965	13.21	2	✚	✚	-	-
303	/	556.6228	13.31	3	✚	✚	-	-
304	/	560.3174	13.31	1	✚	✚	✚	✚
305	/	553.7987	13.56	2	✚	✚	✚	✚
306	/	514.2510	13.59	2	✚	✚	-	✚
307	/	553.3052	13.59	2	✚	✚	✚	✚
308	/	579.7647	13.59	2	✚	✚	✚	✚
309	/	564.2942	13.60	2	✚	✚	✚	✚
310	/	572.2779	13.60	2	✚	✚	✚	✚
311	/	577.8215	13.75	2	✚	✚	-	-
312	/	599.8293	14.00	2	✚	✚	-	-
313	/	535.2696	14.08	1	✚	✚	✚	✚
314	/	587.1737	14.08	1	✚	✚	✚	✚
315	/	550.7959	14.24	2	✚	✚	✚	✚
316	/	542.7990	14.46	2	✚	✚	✚	✚
317	/	545.3081	14.46	2	✚	✚	✚	✚
318	/	555.7896	14.46	2	✚	✚	-	-
319	/	569.6130	14.46	3	✚	✚	✚	✚
320	/	573.2504	14.46	2	✚	✚	✚	✚
321	/	575.3018	14.46	2	✚	✚	✚	✚
322	/	583.6149	14.46	3	✚	✚	-	✚
323	/	594.2725	14.46	2	✚	✚	✚	✚
324	/	539.7992	14.82	2	✚	✚	-	-
325	/	591.8301	14.95	2	✚	✚	-	-
326	/	596.8323	14.97	2	✚	✚	✚	✚
327	/	579.6278	15.28	3	✚	✚	-	-
328	/	585.3025	15.28	1	✚	✚	-	-

329	/	589.3338	15.44	1	✚	✚	✚	✚
330	/	508.2906	15.50	1	✚	✚	✚	✚
331	/	564.2834	15.75	1	✚	✚	✚	✚
332	/	517.6038	16.04	3	✚	✚	-	✚
333	/	546.7834	16.04	2	✚	✚	✚	✚
334	/	588.8368	16.04	2	✚	✚	✚	✚
335	/	587.3193	16.26	1	✚	✚	✚	-
336	/	586.3359	16.42	1	✚	✚	✚	✚
337	/	512.2539	16.68	2	✚	✚	✚	✚
338	/	544.7864	16.68	2	✚	✚	✚	✚
339	/	510.2391	16.77	2	✚	✚	✚	✚
340	/	556.7927	17.20	2	✚	✚	✚	✚
341	/	538.7866	17.27	2	✚	✚	✚	✚
342	/	544.7866	17.43	2	✚	✚	✚	✚
343	/	581.8293	17.50	2	✚	✚	✚	✚
344	/	535.8036	17.75	2	✚	✚	✚	✚
345	/	524.8132	17.94	2	✚	✚	✚	-
346	/	563.8012	17.94	2	✚	✚	✚	✚
347	/	532.2520	18.39	2	✚	✚	-	-
348	/	530.7887	18.89	2	✚	✚	✚	✚
349	**1**	518.2695	18.92	4	✚	-	-	-
350	/	509.7630	19.19	2	✚	✚	✚	✚
351	/	543.3277	19.19	1	✚	✚	-	-
352	/	565.3103	19.19	1	✚	✚	-	-
353	/	529.6020	19.40	3	✚	✚	✚	✚
354	/	515.2510	19.50	2	✚	✚	✚	✚
355	/	512.5736	19.63	3	✚	✚	✚	✚
356	/	530.9570	19.63	3	✚	✚	✚	✚
357	/	538.2836	19.63	3	✚	✚	✚	✚
358	/	543.6046	19.63	3	✚	✚	✚	✚
359	/	548.9296	19.63	3	✚	✚	✚	✚
360	/	581.2635	19.63	2	✚	✚	✚	✚
361	/	507.7421	19.80	2	✚	✚	✚	✚
362	/	511.7312	19.81	2	✚	✚	✚	✚
363	/	518.7344	19.81	2	✚	✚	✚	✚
364	/	526.7177	19.81	2	✚	✚	✚	✚
365	/	534.1999	19.81	2	✚	✚	✚	✚
366	/	571.3244	19.81	1	✚	✚	✚	-
367	/	528.9272	20.10	3	✚	✚	✚	-
368	/	510.9530	20.13	3	✚	✚	-	-
369	/	523.6025	20.13	3	✚	✚	-	-
370	/	528.5910	20.13	3	✚	✚	-	-

371	/	595.6501	20.13	3	✚	✚	-	-
372	/	518.2789	20.15	3	✚	✚	-	-
373	/	513.7482	20.49	2	✚	✚	✚	✚
374	/	598.3134	20.60	2	✚	✚	-	-
375	/	535.6320	21.32	3	✚	✚	-	-
376	/	591.8236	21.95	2	✚	✚	✚	-
377	/	599.3923	22.27	1	✚	✚	✚	✚
378	/	573.3398	23.14	1	✚	✚	✚	✚
379	/	511.2845	23.66	2	✚	✚	✚	✚
380	/	501.7630	24.02	2	✚	✚	✚	✚
381	/	512.7552	24.02	2	✚	✚	✚	✚
382	/	520.7371	24.02	2	✚	✚	✚	✚
383	/	561.3171	24.91	1	✚	✚	✚	✚
384	/	572.3803	25.69	1	✚	✚	✚	✚
385	/	544.3862	26.81	1	✚	✚	✚	✚
386	/	547.7753	27.08	2	✚	✚	✚	✚
387	/	558.7653	27.09	2	✚	✚	✚	✚
388	/	574.2337	27.09	2	✚	✚	✚	✚
389	/	539.7781	28.50	2	✚	✚	✚	✚
390	/	602.3390	8.27	1	✚	✚	✚	✚
391	/	604.3075	9.40	1	✚	✚	✚	✚
392	/	644.3510	10.06	1	✚	✚	✚	✚
393	/	677.3781	10.98	1	✚	✚	✚	✚
394	/	694.4061	11.52	1	✚	✚	✚	✚
395	/	628.3564	12.10	2	✚	✚	✚	✚
396	/	604.2905	12.35	1	✚	✚	✚	✚
397	/	618.3392	12.35	1	✚	✚	✚	✚
398	/	600.2304	12.78		✚	✚	✚	✚
399	/	629.8132	12.78	2	✚	✚	✚	✚
400	/	655.3930	13.03	1	✚	✚	✚	✚
401	/	696.8466	13.03	2	✚	✚	✚	-
402	/	666.8469	13.20	2	✚	✚	✚	✚
403	/	618.3262	13.31	2	✚	✚	-	-
404	/	602.2936	14.24	1	✚	✚	✚	✚
405	/	655.8360	14.50	2	✚	✚	✚	-
406	/	690.8569	14.62	2	✚	✚	-	-
407	/	607.8255	14.80	2	✚	✚	✚	✚
408	/	660.3497	15.00	2	✚	✚	✚	✚
409	/	655.3567	15.28	1	✚	✚	-	✚
410	/	607.2546	15.50	1	✚	✚	✚	✚
411	/	670.3497	15.50	2	✚	✚	✚	✚
412	/	624.3507	15.75	1	✚	✚	✚	✚

413	/	643.3687	16.42	1	✚	✚	✚	✚
414	/	614.6549	16.68	3	✚	✚	-	-
415	/	608.3549	16.74	1	✚	✚	✚	✚
416	/	654.3494	16.74	2	✚	✚	-	-
417	/	644.8290	16.75	2	✚	✚	✚	-
418	/	602.3176	17.10	3	✚	✚	-	✚
419	/	621.9774	17.10	3	✚	✚	✚	-
420	/	662.3516	17.34	2	✚	✚	✚	✚
421	/	609.8244	17.75	2	✚	✚	✚	✚
422	/	647.3307	17.75	1	✚	✚	✚	✚
423	/	654.3328	17.94	2	✚	✚	✚	✚
424	/	601.3122	18.39	2	✚	✚	✚	✚
425	/	600.9833	18.89	3	✚	✚	-	-
426	/	649.3496	18.89	3	✚	✚	-	-
427	/	672.3542	18.90	2	✚	✚	✚	✚
428	/	698.3878	19.19	1	✚	✚	-	-
429	/	607.2905	19.63	1	✚	✚	✚	✚
430	/	669.3605	19.63	2	✚	✚	-	-
431	/	649.3000	19.82	1	✚	✚	✚	✚
432	/	672.8430	20.00	2	✚	✚	✚	✚
433	/	648.3511	20.10	2	✚	✚	✚	✚
434	/	600.3867	20.13	1	✚	✚	✚	✚
435	/	635.3197	20.13	1	✚	✚	-	-
436	/	688.4038	20.13	1	✚	✚	✚	✚
437	/	683.8020	20.49	2	✚	✚	✚	✚
438	/	664.8443	20.70	2	✚	✚	✚	✚
439	/	661.3627	20.80	2	✚	✚	-	-
440	/	621.3747	21.95	1	✚	✚	✚	✚
441	/	646.7862	21.95	2	✚	✚	✚	✚
442	/	656.8466	21.95	2	✚	✚	✚	✚
443	/	603.2689	22.63	2	✚	✚	✚	✚
444	/	649.3232	22.63	2	✚	✚	✚	✚
445	/	623.8404	23.30	2	✚	✚	-	-
446	/	641.3310	23.36	2	✚	✚	✚	✚
447	/	648.8497	23.36	2	✚	✚	✚	✚
448	/	670.4318	23.36	1	✚	✚	✚	✚
449	**2**	675.6632	23.36	3	✚	-	-	-
450	/	656.4151	23.66	1	✚	✚	-	-
451	/	676.8740	26.90	2	✚	✚	-	-
452	/	745.3828	5.59		✚	✚	✚	✚
453	/	767.4245	8.27	1	✚	✚	✚	-
454	/	771.3333	10.39	1	✚	✚	✚	✚

455	/	785.4468	10.98	1	+	+	+	-
456	/	726.3605	11.86	1	+	+	+	+
457	/	780.7086	11.86	3	+	+	-	-
458	/	773.3523	12.10		+	+	+	+
459	/	748.3914	12.40	1	+	+	+	+
460	/	731.8613	12.90	2	+	+	-	+
461	/	762.8742	13.03	2	+	+	-	+
462	/	783.4554	13.03	1	+	+	+	+
463	/	723.3107	13.49	1	+	+	+	+
464	/	746.8769	13.70	2	+	+	-	-
465	/	763.8432	13.80	2	+	+	-	-
466	/	724.3671	13.88	2	+	+	-	+
467	/	766.7067	13.88	3	+	+	+	+
468	/	708.6879	14.08	3	+	+	+	+
469	/	718.2710	14.08	1	+	+	+	+
470	/	703.3402	14.46	2	+	+	+	+
471	/	761.0394	14.46	3	+	+	+	+
472	/	778.3712	14.88	2	+	+	+	+
473	/	749.0318	14.89	3	+	+	+	+
474	/	753.8639	14.89	2	+	+	+	+
475	/	767.7089	14.89	3	+	+	+	-
476	/	755.7079	15.33	3	+	+	+	+
477	/	710.8729	15.35	2	+	+	+	+
478	/	745.8657	15.95	2	+	+	-	+
479	/	775.9012	16.04	2	+	+	-	+
480	/	798.9020	16.04	2	+	+	-	+
481	/	733.3679	16.20	2	+	+	-	-
482	/	730.3692	16.42	2	+	+	+	+
483	/	760.8886	16.89	2	+	+	-	-
484	/	732.3417	17.04	1	+	+	+	+
485	/	723.7094	17.26	3	+	+	+	-
486	/	724.0438	17.34	3	+	+	+	-
487	/	718.7115	18.11	3	+	+	+	-
488	/	792.8801	18.34	2	+	+	+	+
489	/	737.6915	18.80	3	+	+	-	-
490	/	708.3562	18.89	2	+	+	+	+
491	/	713.0401	19.05	3	+	+	+	-
492	/	793.8986	19.40	2	+	+	+	+
493	/	795.9306	19.61	2	+	+	+	+
494	/	785.8968	19.81	2	+	+	+	+
495	/	765.9196	20.10	2	+	+	+	-
496	/	726.3114	20.13	2	+	+	+	+

497	/	765.9238	20.13	2	✚	✚	-	-
498	/	776.9109	20.13	2	✚	✚	-	-
499	/	784.8969	20.13	2	✚	✚	✚	✚
500	/	761.0683	20.49	3	✚	✚	-	-
501	/	705.8646	21.52	2	✚	✚	✚	-
502	/	753.3849	21.52	2	✚	✚	-	-
503	/	781.9374	21.52	2	✚	✚	✚	✚
504	/	728.4091	22.27	2	✚	✚	-	-
505	/	781.9127	22.43	2	✚	✚	✚	✚
506	**4**	766.6952	23.36	3	✚	-	-	-
507	**5**	774.0218	23.36	3	✚	-	-	-
508	**6**	784.3392	23.36	3	✚	-	-	-
509	/	786.4556	23.66	1	✚	✚	-	-
510	**3**	761.3672	24.24	3	✚	-	-	-
511	/	757.4285	24.97	1	✚	✚	✚	-
512	/	868.4834	10.62	1	✚	✚	✚	✚
513	/	800.3909	11.86	2	✚	✚	✚	-
514	/	811.4634	12.10	1	✚	✚	✚	✚
515	/	811.4623	12.27	1	✚	✚	✚	✚
516	/	928.4948	12.27	1	✚	✚	✚	✚
517	/	811.4610	12.30	1	✚	✚	✚	✚
518	/	883.4320	12.40	2	✚	✚	-	✚
519	/	937.5318	13.03	1	✚	✚	-	-
520	/	811.4614	13.13	1	✚	✚	✚	-
521	/	811.4615	13.20	1	✚	✚	✚	-
522	/	804.8810	13.59	2	✚	✚	✚	✚
523	/	854.4121	14.46	2	✚	✚	✚	✚
524	/	874.9170	14.46	2	✚	✚	-	✚
525	/	892.5215	14.62	1	✚	✚	✚	✚
526	/	868.9328	14.88	2	✚	✚	-	-
527	/	845.9125	15.33	2	✚	✚	✚	✚
528	/	813.4302	15.75	1	✚	✚	✚	✚
529	/	823.5000	15.75	1	✚	✚	✚	-
530	/	816.4244	16.24	2	✚	✚	✚	✚
531	/	898.5327	16.42	1	✚	✚	✚	✚
532	/	825.9096	16.49	2	✚	✚	-	-
533	/	921.4785	16.68	2	✚	✚	-	-
534	/	852.4311	17.04	1	✚	✚	✚	✚
535	/	834.7436	17.43	3	✚	✚	✚	✚
536	/	934.4512	17.75	1	✚	✚	✚	✚
537	/	874.7801	17.92	3	✚	✚	✚	✚
538	/	804.3942	18.11	2	✚	✚	-	✚

539	/	811.7179	18.34	3	✚	✚	✚	✚
540	/	900.9699	18.89	2	✚	✚	-	-
541	/	829.4146	19.19	2	✚	✚	-	-
542	/	824.4354	19.63	2	✚	✚	✚	✚
543	/	892.9678	20.00	2	✚	✚	-	-
544	/	841.4766	20.13	1	✚	✚	✚	✚
545	/	821.4173	20.49	2	✚	✚	-	-
546	/	850.4249	21.02	3	✚	✚	✚	✚
547	/	976.5327	21.02	1	✚	✚	-	-
548	/	821.3904	21.20	1	✚	✚	✚	✚
549	/	802.9475	21.40	2	✚	✚	-	-
550	/	845.4240	22.27	3	✚	✚	✚	✚
551	/	960.5383	22.63	1	✚	✚	-	-
552	/	844.4641	23.36	1	✚	✚	✚	-
553	/	926.5504	23.66	1	✚	✚	✚	✚
554	**7**	854.0603	27.08	3	✚	-	-	-
555	/	874.5250	31.78	1	✚	✚	✚	✚
556	/	1008.4980	12.55	1	✚	✚	✚	-
557	/	1027.4908	13.59	1	✚	✚	-	✚
558	/	1105.5997	13.59	1	✚	✚	✚	✚
559	/	1149.5944	14.46	1	✚	✚	✚	✚
560	/	1078.5911	14.88	1	✚	✚	-	-
561	/	1192.6607	15.00	1	✚	✚	✚	✚
562	/	1092.5548	16.04	1	✚	✚	✚	✚
563	/	1088.5613	17.34	1	✚	✚	✚	✚
564	/	1018.5114	19.19	1	✚	✚	✚	✚
565	/	1029.4871	19.50	1	✚	✚	✚	✚
566	/	1014.4776	19.81	1	✚	✚	✚	✚
567	**4**	1150.0642	23.40	2	✚	-	-	-
568	/	1021.5555	23.66		✚	✚	✚	✚
569	/	1094.5447	27.08	1	✚	✚	✚	✚

Notes: The number in the first column is the same as Fig. 1. Candidates were firstly screened from BPC of standard substance of donkey-hide. Subsequently, they further confirmed by comparing EIC of four animal species. "✚" indicates that the marker candidate was detected by UPLC-Q-TOF-MS. "-" indicates that the marker candidate was not detected. These marker candidates showed two/three/four charges and were deduced to be peptides.

Supplementary Table S2

Markers selection for horse-hide gelatin from peptide fragments based on trypsin digestion followed by LC-Q-TOF-MS analysis.

Horse marker No.	m/z	RT (min)	Charge	Donkey	Horse	Cattle	Pig
/	189.1288	5.31	1	✚	✚	✚	✚
/	216.6198	8.26	2	✚	✚	✚	✚
/	219.1404	10.98	1	✚	✚	✚	✚
/	223.1141	8.27	1	✚	✚	✚	✚
/	224.1352	31.00	1	✚	✚	✚	✚
/	228.6384	5.31	2	✚	✚	✚	✚

/	229.6280	12.09	2	✚	✚	✚	✚
/	231.1762	10.44	1	✚	✚	✚	✚
/	231.1771	7.77		✚	✚	✚	✚
/	233.1565	5.55	1	✚	✚	✚	✚
/	234.1024	22.27		✚	✚	✚	✚
/	236.6544	9.34	2	✚	✚	✚	✚
/	237.6260	10.08	2	✚	✚	✚	✚
/	237.6447	5.31	2	✚	✚	✚	✚
/	242.1565	10.08	1	✚	✚	✚	✚
/	243.1408	15.75	1	✚	✚	✚	✚
/	245.1925	14.24	1	✚	✚	✚	✚
/	245.1925	15.38	1	✚	✚	✚	✚
/	249.6631	10.08	2	✚	✚	✚	✚
/	250.1855	32.78	1	✚	✚	✚	✚
/	258.1766	10.10	2	✚	✚	✚	✚
/	260.1678	9.58	1	✚	✚	✚	✚
/	260.2043	6.27	1	✚	✚	✚	✚
/	265.1621	13.32	1	✚	✚	✚	✚
/	267.6266	6.27	2	✚	✚	✚	✚
/	270.2001	10.08	1	✚	✚	✚	✚
/	272.1795	9.40	1	✚	✚	✚	✚
/	272.6535	12.27	2	✚	✚	✚	✚
/	274.1954	10.08	1	✚	✚	✚	✚
/	276.1632	7.57	1	✚	✚	✚	✚
/	277.1257	9.74	1	✚	✚	✚	✚
/	278.6662	12.50	2	✚	✚	✚	✚
/	279.1418	17.53	1	✚	✚	✚	✚
/	279.1777	18.20	1	✚	✚	✚	✚
/	281.1213	10.08	1	✚	✚	✚	✚
/	281.1566	5.59	1	✚	✚	✚	✚
/	283.8095	12.27	2	✚	✚	✚	✚
/	288.2009	20.49		✚	✚	✚	✚
/	288.2116	6.30		✚	✚	✚	✚
/	290.1784	6.30		✚	✚	✚	✚
/	292.6630	12.27	2	✚	✚	✚	✚
/	295.1730	10.98	1	✚	✚	✚	✚
/	295.1740	10.08	1	✚	✚	✚	✚
/	297.6402	12.50	2	✚	✚	✚	✚
/	300.6175	12.78	2	✚	✚	✚	✚
/	301.1832	6.30	2	✚	✚	✚	✚
/	301.6758	8.27	2	✚	✚	✚	✚
/	301.6768	14.24	2	✚	✚	✚	✚

/	302.1801	10.08	1	✚	✚	✚	✚
/	302.2159	19.22	1	✚	✚	✚	✚
/	302.2166	18.21	1	✚	✚	✚	✚
/	303.6207	6.30	1	✚	✚	✚	✚
/	305.6956	11.52	2	✚	✚	✚	✚
/	306.1484	23.08	2	✚	✚	✚	✚
/	306.1739	6.30		✚	✚	✚	✚
/	309.6738	12.35	2	✚	✚	✚	✚
/	312.8329	16.42	3	✚	✚	✚	✚
/	314.6831	12.07	2	✚	✚	✚	✚
/	314.7013	11.46	2	✚	✚	✚	-
/	316.2312	19.63	1	✚	✚	✚	✚
/	317.1910	6.30	3	✚	✚	✚	✚
/	320.1733	14.88	1	✚	✚	✚	✚
/	320.1866	8.27	1	✚	✚	✚	-
/	320.2031	12.78	3	✚	✚	✚	✚
/	321.1797	13.31	2	✚	✚	✚	-
/	321.1817	5.59	1	✚	✚	✚	✚
/	322.6805	10.06	2	✚	✚	✚	✚
/	322.8205	12.27	3	✚	✚	✚	✚
/	323.1587	6.30		✚	✚	✚	✚
/	323.4940	6.30		✚	✚	✚	✚
/	324.1811	5.59	2	✚	✚	✚	✚
/	325.6760	8.17	2	✚	✚	-	-
/	327.8098	12.27	3	✚	✚	✚	✚
/	329.1909	6.30	2	✚	✚	✚	✚
/	329.2007	5.31	1	✚	✚	✚	✚
/	329.2282	15.00	2	✚	✚	✚	✚
/	330.1635	19.95	1	✚	✚	✚	✚
/	330.2011	12.78	2	✚	✚	✚	✚
/	332.2266	14.24	1	✚	✚	✚	✚
/	332.2273	18.89	1	✚	✚	✚	✚
/	335.1611	21.76	3	✚	✚	✚	✚
/	337.1392	20.13	2	✚	✚	-	-
/	338.8360	21.02		✚	✚	-	-
/	339.1935	10.87	2	✚	✚	✚	✚
/	339.6729	11.52	2	✚	✚	✚	✚
/	343.2051	12.27		✚	✚	✚	✚
/	344.2632	20.16	1	✚	✚	✚	✚
/	345.1529	9.63	1	✚	✚	✚	✚
/	345.2333	5.31	1	✚	✚	✚	✚
/	347.2014	7.77	1	✚	✚	✚	✚

/	347.7072	11.46	2	✚	✚	✚	✚
/	350.1806	9.63	1	✚	✚	✚	✚
/	350.6348	14.08	2	✚	✚	✚	✚
/	353.4912	14.88	3	✚	✚	✚	✚
/	359.2392	10.08	1	✚	✚	✚	✚
/	359.6404	14.08	2	✚	✚	✚	✚
/	365.8721	8.27		✚	✚	✚	-
/	366.2176	17.35	2	✚	✚	✚	✚
/	369.2061	13.59	3	✚	✚	✚	✚
/	369.7033	6.30	2	✚	✚	✚	✚
/	371.2385	10.98	1	✚	✚	✚	✚
/	371.7236	16.41	2	✚	✚	✚	✚
/	372.2159	10.36	2	✚	✚	✚	✚
/	372.6956	5.59	2	✚	✚	✚	✚
/	372.8516	14.88	3	✚	✚	-	-
/	373.2899	6.30	1	✚	✚	✚	✚
/	374.2389	18.89	1	✚	✚	✚	✚
/	374.7011	12.41	2	✚	✚	✚	✚
/	374.8536	14.46	3	✚	✚	✚	✚
/	375.2342	7.77	1	✚	✚	✚	✚
/	377.1926	13.59	1	✚	✚	✚	✚
/	377.1953	11.46	1	✚	✚	✚	✚
/	377.8407	14.88	3	✚	✚	-	-
/	378.6137	14.08	2	✚	✚	✚	✚
/	379.2181	24.94	2	✚	✚	✚	✚
/	379.7035	10.98	2	✚	✚	✚	✚
/	379.8415	14.46	3	✚	✚	✚	✚
/	381.8550	13.59	3	✚	✚	✚	✚
/	382.5030	14.46	3	✚	✚	✚	✚
/	383.7419	15.00	2	✚	✚	✚	-
/	384.2149	8.64	2	✚	✚	✚	-
/	385.2063	8.27	2	✚	✚	✚	✚
/	385.2297	10.08	1	✚	✚	✚	✚
/	385.2661	9.40	1	✚	✚	✚	✚
/	385.5505	13.59	3	✚	✚	-	-
**1**	386.2108	10.36	2	-	✚	-	-
/	386.8436	13.59	3	✚	✚	✚	✚
/	387.8462	19.63	3	✚	✚	✚	✚
/	388.2293	6.30		✚	✚	✚	✚
/	388.2296	12.27		✚	✚	✚	✚
/	389.2488	11.01	1	✚	✚	✚	✚
/	390.2222	10.60	2	✚	✚	✚	✚

/	390.8836	12.91	3	✚	✚	-	-
/	391.1910	5.31	1	✚	✚	✚	✚
/	391.2092	18.32	1	✚	✚	✚	✚
/	392.2315	13.05	2	✚	✚	✚	✚
/	392.9530	20.13	4	✚	✚	-	-
/	393.2091	9.40	2	✚	✚	✚	✚
/	393.2267	11.01	2	✚	✚	✚	-
/	393.7321	23.66	2	✚	✚	-	-
/	395.6893	8.94	2	✚	✚	✚	✚
/	396.2707	14.62	1	✚	✚	✚	✚
/	396.6962	20.13	4	✚	✚	✚	-
/	398.1658	6.30		✚	✚	-	✚
/	398.2194	10.08		✚	✚	✚	-
/	398.2272	15.00	3	✚	✚	✚	✚
/	398.2504	14.18	1	✚	✚	✚	✚
/	399.2711	17.34	1	✚	✚	✚	✚
/	400.2297	9.40	1	✚	✚	✚	✚
/	400.2298	12.78	2	✚	✚	✚	✚
/	402.2567	10.08	1	✚	✚	✚	✚
/	402.5164	22.63	3	✚	✚	✚	✚
/	403.2657	15.00	1	✚	✚	✚	✚
/	403.2657	16.04	1	✚	✚	-	-
/	404.2236	9.40	1	✚	✚	✚	✚
/	404.4604	19.63	4	✚	✚	✚	✚
/	405.2085	10.36		✚	✚	-	-
/	405.2186	9.63	2	✚	✚	✚	✚
/	406.2352	13.25	2	✚	✚	✚	-
/	406.2358	12.21	2	✚	✚	✚	✚
/	407.2195	15.76	2	✚	✚	✚	✚
/	407.9573	19.63	4	✚	✚	✚	✚
/	410.8747	15.00	3	✚	✚	✚	✚
/	411.2322	10.98	1	✚	✚	✚	✚
/	411.6992	19.63	4	✚	✚	✚	✚
/	412.2536	16.04	2	✚	✚	✚	✚
/	412.2540	15.75	2	✚	✚	✚	✚
/	413.2410	14.18	2	✚	✚	✚	✚
/	413.2866	21.76	1	✚	✚	✚	✚
/	414.2108	14.52	2	✚	✚	-	-
/	414.2413	14.88	3	✚	✚	✚	✚
/	414.2457	12.27	1	✚	✚	✚	✚
/	415.2447	22.63	1	✚	✚	✚	✚
/	415.8626	15.00	3	✚	✚	✚	✚

/	418.2195	17.95	1	+	+	+	+
/	418.7339	10.36	2	+	+	+	+
/	420.1979	13.01	1	+	+	+	+
/	421.2412	12.45	2	+	+	+	+
/	421.2432	20.13	2	+	+	+	+
/	421.2612	15.00	2	+	+	+	+
/	421.7331	9.63	2	+	+	+	+
/	422.2127	5.31	1	+	+	+	+
/	426.7299	8.94	2	+	+	+	+
/	427.7383	11.01	2	+	+	-	-
/	427.7393	10.98	2	+	+	-	-
/	429.2457	22.30	1	+	+	+	+
/	429.2826	21.01	1	+	+	+	+
/	430.2411	12.78	1	+	+	+	+
/	430.2775	20.63	1	+	+	+	+
/	431.2253	13.59	2	+	+	+	+
/	431.2478	6.30	1	+	+	+	+
/	431.5276	21.95	3	+	+	+	+
/	432.2315	8.27	1	+	+	+	+
/	434.2619	17.34	2	+	+	+	+
/	434.7471	10.81	2	+	+	+	+
/	437.7673	31.77	2	+	+	+	+
/	441.2521	20.49	2	+	+	+	+
/	441.7730	18.54	2	+	+	+	+
/	442.7441	12.27	2	+	+	+	+
/	443.2353	6.30	1	+	+	+	+
/	444.2303	10.08	2	+	+	+	+
/	444.2936	20.49	1	+	+	+	+
/	445.2527	7.80	1	+	+	+	+
/	446.2717	8.27	1	+	+	+	+
/	446.2722	16.69	1	+	+	+	+
/	446.5774	19.63	3	+	+	-	-
/	446.7472	11.52	2	+	+	+	+
/	446.7659	14.62	2	+	+	+	+
/	447.2085	13.59	1	+	+	+	+
/	447.2105	6.30	1	+	+	+	+
/	447.2542	14.60	1	+	+	+	+
/	448.2467	10.36	2	+	+	+	+
/	449.7704	16.48	2	+	+	+	+
/	451.2280	22.27	1	+	+	+	+
/	453.2203	12.27		+	+	+	+
/	454.7644	13.80	2	+	+	+	+

/	455.2042	19.61	1	✚	✚	✚	✚
**2**	455.2539	10.90	2	-	✚	-	-
/	456.2434	8.94	2	✚	✚	✚	✚
/	456.2456	13.15	2	✚	✚	✚	✚
/	456.2925	23.26	1	✚	✚	✚	✚
/	457.2030	14.88	2	✚	✚	✚	✚
/	457.7509	12.27	2	✚	✚	✚	✚
/	458.2476	12.10	2	✚	✚	✚	✚
/	459.1892	12.27	2	✚	✚	✚	✚
/	459.2278	19.63	3	✚	✚	✚	✚
/	459.2678	10.98	1	✚	✚	✚	✚
/	459.2685	5.59	1	✚	✚	✚	✚
/	459.2935	21.96	1	✚	✚	✚	✚
/	460.4769	18.89	4	✚	✚	-	-
/	461.2554	11.46	2	✚	✚	-	-
/	462.2302	6.30	1	✚	✚	✚	✚
/	462.2472	19.19	1	✚	✚	✚	✚
/	464.2263	13.90	1	✚	✚	✚	✚
/	464.4752	18.89	4	✚	✚	-	-
/	464.7522	12.26	2	✚	✚	✚	✚
/	465.2464	14.88	1	✚	✚	✚	✚
/	467.7294	17.95	2	✚	✚	✚	✚
/	467.7517	11.01	2	✚	✚	✚	✚
/	468.2369	11.52	1	✚	✚	✚	✚
/	469.2535	10.08	2	✚	✚	-	-
/	469.2711	13.03	2	✚	✚	-	-
/	471.7388	12.84	2	✚	✚	✚	✚
/	472.2880	13.31	1	✚	✚	✚	✚
/	472.2893	21.02	1	✚	✚	✚	✚
/	472.2992	9.40	1	✚	✚	✚	✚
/	472.5596	18.89	3	✚	✚	✚	✚
/	473.2079	18.21	2	✚	✚	✚	✚
/	473.2346	5.59		✚	✚	✚	-
/	474.2428	11.52	1	✚	✚	✚	✚
/	474.2429	10.08	1	✚	✚	✚	✚
/	474.2679	12.27	2	✚	✚	✚	✚
/	474.2682	12.27	1	✚	✚	✚	✚
/	474.2799	5.31	1	✚	✚	✚	✚
/	475.7502	10.57	2	✚	✚	✚	✚
/	476.2273	18.89	1	✚	✚	✚	✚
/	476.2476	12.35	2	✚	✚	✚	✚
/	478.2575	16.50	2	✚	✚	-	✚

/	478.7565	13.59	2	✚	✚	✚	✚
/	479.2622	17.50	1	✚	✚	✚	✚
/	479.7979	12.78	2	✚	✚	✚	✚
/	480.2565	21.76	1	✚	✚	✚	✚
/	480.7737	22.63	2	✚	✚	-	-
/	484.2314	6.30	2	✚	✚	✚	✚
/	486.3042	14.60	1	✚	✚	✚	✚
/	486.3052	14.62	1	✚	✚	✚	✚
/	488.7716	21.02	2	✚	✚	-	-
/	490.2600	5.59		✚	✚	✚	✚
/	491.7648	22.63	2	✚	✚	-	-
/	492.5855	12.10	3	✚	✚	-	-
/	493.2424	19.19	1	✚	✚	✚	✚
/	498.7538	9.63	2	✚	✚	✚	✚
/	499.2625	7.80	1	✚	✚	✚	✚
/	499.2998	14.24	1	✚	✚	-	-
/	499.7645	21.01	2	✚	✚	✚	✚
/	501.2429	6.30	1	✚	✚	✚	✚
/	501.7630	24.02	2	✚	✚	✚	✚
/	504.7541	12.50	2	✚	✚	✚	-
/	507.7421	19.80	2	✚	✚	✚	✚
/	508.2906	15.50	1	✚	✚	✚	✚
/	509.7630	19.19	2	✚	✚	✚	✚
/	510.2391	16.77	2	✚	✚	✚	✚
/	510.9530	20.13	3	✚	✚	-	-
/	511.2845	23.66	2	✚	✚	✚	✚
/	511.7312	19.81	2	✚	✚	✚	✚
/	512.2539	16.68	2	✚	✚	✚	✚
/	512.5736	19.63	3	✚	✚	✚	✚
/	512.7552	24.02	2	✚	✚	✚	✚
/	513.7482	20.49	2	✚	✚	✚	✚
/	514.2510	13.59	2	✚	✚	-	✚
/	515.2510	19.50	2	✚	✚	✚	✚
/	515.3429	10.06	1	✚	✚	✚	✚
/	517.6038	16.04	3	✚	✚	-	✚
/	518.2789	20.15	3	✚	✚	-	-
/	518.7344	19.81	2	✚	✚	✚	✚
/	520.7371	24.02	2	✚	✚	✚	✚
/	520.9586	20.16	3	-	✚	✚	-
/	523.6025	20.13	3	✚	✚	-	-
/	524.7756	11.14	2	-	✚	✚	-
/	524.8132	17.94	2	✚	✚	✚	-

/	526.2750	5.31	1	✚	✚	✚	✚
/	526.7177	19.81	2	✚	✚	✚	✚
/	527.2957	12.10	1	✚	✚	✚	✚
/	528.5910	20.13	3	✚	✚	-	-
/	528.9272	20.10	3	✚	✚	✚	-
/	529.6020	19.40	3	✚	✚	✚	✚
/	530.7887	18.89	2	✚	✚	✚	✚
/	530.9570	19.63	3	✚	✚	✚	✚
/	531.2907	12.27	1	✚	✚	✚	✚
/	531.7855	12.78	2	✚	✚	✚	✚
/	531.7965	13.21	2	✚	✚	-	-
/	532.2520	18.39	2	✚	✚	-	-
/	534.1999	19.81	2	✚	✚	✚	✚
/	534.2432	6.30	1	✚	✚	✚	✚
/	535.2696	14.08	1	✚	✚	✚	✚
/	535.6320	21.32	3	✚	✚	-	-
/	535.8036	17.75	2	✚	✚	✚	✚
/	536.7798	12.35	2	✚	✚	✚	-
/	538.2274	12.65	2	✚	✚	✚	✚
/	538.2836	19.63	3	✚	✚	✚	✚
/	538.7866	17.27	2	✚	✚	✚	✚
/	539.7781	28.50	2	✚	✚	✚	✚
/	539.7992	14.82	2	✚	✚	-	-
/	542.7990	14.46	2	✚	✚	✚	✚
/	543.3277	19.19	1	✚	✚	-	-
/	543.6046	19.63	3	✚	✚	✚	✚
/	544.2973	12.40	1	✚	✚	✚	✚
/	544.3862	26.81	1	✚	✚	✚	✚
/	544.7864	16.68	2	✚	✚	✚	✚
/	544.7866	17.43	2	✚	✚	✚	✚
/	545.3081	14.46	2	✚	✚	✚	✚
/	546.7834	16.04	2	✚	✚	✚	✚
/	547.7753	27.08	2	✚	✚	✚	✚
/	548.9296	19.63	3	✚	✚	✚	✚
/	550.7959	14.24	2	✚	✚	✚	✚
/	552.6142	12.58	3	✚	✚	✚	✚
**3**	552.7825	15.98	2	-	✚	-	-
/	553.3052	13.59	2	✚	✚	✚	✚
/	553.7987	13.56	2	✚	✚	✚	✚
/	555.7896	14.46	2	✚	✚	-	-
/	556.3212	12.55	1	✚	✚	✚	✚
/	556.6228	13.31	3	✚	✚	-	-

/	556.7927	17.20	2	✚	✚	✚	✚
/	558.3128	5.59		✚	✚	✚	✚
/	558.7653	27.09	2	✚	✚	✚	✚
/	560.3174	13.31	1	✚	✚	✚	✚
/	561.3171	24.91	1	✚	✚	✚	✚
/	562.2794	12.78	1	✚	✚	✚	✚
/	563.8012	17.94	2	✚	✚	✚	✚
/	564.2834	15.75	1	✚	✚	✚	✚
/	564.2942	13.60	2	✚	✚	✚	✚
/	565.3103	19.19	1	✚	✚	-	-
/	568.2868	6.30	1	✚	✚	✚	✚
/	569.6130	14.46	3	✚	✚	✚	✚
/	569.7932	10.06	2	✚	✚	✚	✚
/	570.3125	12.10	1	✚	✚	✚	✚
/	571.2687	13.00	1	-	✚	✚	✚
/	571.2819	12.27	1	✚	✚	✚	✚
/	571.3244	19.81	1	✚	✚	✚	-
/	572.2779	13.60	2	✚	✚	✚	✚
/	572.3803	25.69	1	✚	✚	✚	✚
/	573.2504	14.46	2	✚	✚	✚	✚
/	573.2644	5.31	1	✚	✚	✚	✚
/	573.3398	23.14	1	✚	✚	✚	✚
/	574.2337	27.09	2	✚	✚	✚	✚
/	575.3018	14.46	2	✚	✚	✚	✚
/	577.8215	13.75	2	✚	✚	-	-
/	578.2752	5.31	1	✚	✚	✚	✚
/	578.3000	12.27	2	✚	✚	✚	✚
/	578.3056	12.53	1	✚	✚	✚	✚
/	579.6278	15.28	3	✚	✚	-	-
/	579.7647	13.59	2	✚	✚	✚	✚
/	581.2635	19.63	2	✚	✚	✚	✚
/	581.3193	11.52	1	✚	✚	✚	✚
/	581.8293	17.50	2	✚	✚	✚	✚
/	583.6149	14.46	3	✚	✚	-	✚
/	583.8135	12.10	2	✚	✚	✚	✚
/	584.3129	6.30		✚	✚	✚	✚
/	585.3025	15.28	1	✚	✚	-	-
/	586.3359	16.42	1	✚	✚	✚	✚
/	587.1737	14.08	1	✚	✚	✚	✚
/	587.3193	16.26	1	✚	✚	✚	-
/	588.8368	16.04	2	✚	✚	✚	✚
/	589.2899	12.40	3	✚	✚	-	✚

/	589.3338	15.44	1	✚	✚	✚	✚
/	591.8236	21.95	2	✚	✚	✚	-
/	591.8301	14.95	2	✚	✚	-	-
/	594.2725	14.46	2	✚	✚	✚	✚
/	595.6501	20.13	3	✚	✚	-	-
/	596.3298	12.10	1	✚	✚	✚	✚
/	596.8323	14.97	2	✚	✚	✚	✚
/	598.3134	20.60	2	✚	✚	-	-
/	599.3923	22.27	1	✚	✚	✚	✚
/	599.8293	14.00	2	✚	✚	-	-
/	600.2304	12.78		✚	✚	✚	✚
/	600.3867	20.13	1	✚	✚	✚	✚
/	600.9833	18.89	3	✚	✚	-	-
/	601.3122	18.39	2	✚	✚	✚	✚
/	602.2936	14.24	1	✚	✚	✚	✚
/	602.3176	17.10	3	✚	✚	-	✚
/	602.3390	8.27	1	✚	✚	✚	✚
/	603.2689	22.63	2	✚	✚	✚	✚
/	604.2905	12.35	1	✚	✚	✚	✚
/	604.3075	9.40	1	✚	✚	✚	✚
/	607.2546	15.50	1	✚	✚	✚	✚
/	607.2905	19.63	1	✚	✚	✚	✚
/	607.8255	14.80	2	✚	✚	✚	✚
/	608.3549	16.74	1	✚	✚	✚	✚
/	609.8244	17.75	2	✚	✚	✚	✚
/	614.6549	16.68	3	✚	✚	-	-
/	618.3262	13.31	2	✚	✚	-	-
/	618.3392	12.35	1	✚	✚	✚	✚
/	621.3747	21.95	1	✚	✚	✚	✚
/	621.9774	17.10	3	✚	✚	✚	-
/	623.8404	23.30	2	✚	✚	-	-
/	624.3507	15.75	1	✚	✚	✚	✚
/	628.3564	12.10	2	✚	✚	✚	✚
/	629.8132	12.78	2	✚	✚	✚	✚
/	635.3197	20.13	1	✚	✚	-	-
/	641.3310	23.36	2	✚	✚	✚	✚
/	643.3687	16.42	1	✚	✚	✚	✚
/	644.3510	10.06	1	✚	✚	✚	✚
/	644.8290	16.75	2	✚	✚	✚	-
/	646.7862	21.95	2	✚	✚	✚	✚
/	647.3307	17.75	1	✚	✚	✚	✚
/	648.3511	20.10	2	✚	✚	✚	✚

/	648.8497	23.36	2	+	+	+	+
/	649.3000	19.82	1	+	+	+	+
/	649.3232	22.63	2	+	+	+	+
/	649.3496	18.89	3	+	+	-	-
/	654.3328	17.94	2	+	+	+	+
/	654.3494	16.74	2	+	+	-	-
/	655.3567	15.28	1	+	+	-	+
/	655.3930	13.03	1	+	+	+	+
/	655.8360	14.50	2	+	+	+	-
/	656.4151	23.66	1	+	+	-	-
/	656.8466	21.95	2	+	+	+	+
/	660.3497	15.00	2	+	+	+	+
/	661.3627	20.80	2	+	+	-	-
/	662.3516	17.34	2	+	+	+	+
/	664.8443	20.70	2	+	+	+	+
/	666.8469	13.20	2	+	+	+	+
/	669.3605	19.63	2	+	+	-	-
/	670.3497	15.50	2	+	+	+	+
/	670.4318	23.36	1	+	+	+	+
/	672.3542	18.90	2	+	+	+	+
/	672.8430	20.00	2	+	+	+	+
/	676.8740	26.90	2	+	+	-	-
/	677.3781	10.98	1	+	+	+	+
/	683.8020	20.49	2	+	+	+	+
/	688.4038	20.13	1	+	+	+	+
/	690.3999	25.69	1	-	+	+	-
/	690.8569	14.62	2	+	+	-	-
/	694.4061	11.52	1	+	+	+	+
/	696.8466	13.03	2	+	+	+	-
/	698.3878	19.19	1	+	+	-	-
/	703.3402	14.46	2	+	+	+	+
/	705.8646	21.52	2	+	+	+	-
/	708.3562	18.89	2	+	+	+	+
/	708.6879	14.08	3	+	+	+	+
/	710.8729	15.35	2	+	+	+	+
/	713.0401	19.05	3	+	+	+	-
**4**	716.4201	27.31	1	-	+	-	-
/	718.2710	14.08	1	+	+	+	+
/	718.7115	18.11	3	+	+	+	-
/	723.3107	13.49	1	+	+	+	+
/	723.7094	17.26	3	+	+	+	-
/	724.0438	17.34	3	+	+	+	-

/	724.3671	13.88	2	+	+	-	+
/	726.3114	20.13	2	+	+	+	+
/	726.3605	11.86	1	+	+	+	+
/	728.4091	22.27	2	+	+	-	-
/	730.3692	16.42	2	+	+	+	+
/	731.8613	12.90	2	+	+	-	+
/	732.3417	17.04	1	+	+	+	+
/	733.3679	16.20	2	+	+	-	-
/	737.6915	18.80	3	+	+	-	-
/	745.3828	5.59		+	+	+	+
/	745.8657	15.95	2	+	+	-	+
/	745.8669	15.33	2	-	+	+	-
/	746.8769	13.70	2	+	+	-	-
/	748.3914	12.40	1	+	+	+	+
/	749.0318	14.89	3	+	+	+	+
/	753.3849	21.52	2	+	+	-	-
/	753.8639	14.89	2	+	+	+	+
/	755.7079	15.33	3	+	+	+	+
/	757.4285	24.97	1	+	+	+	-
/	760.8886	16.89	2	+	+	-	-
/	761.0394	14.46	3	+	+	+	+
/	761.0683	20.49	3	+	+	-	-
/	762.3766	22.94	3	-	+	-	+
/	762.8742	13.03	2	+	+	-	+
/	763.8432	13.80	2	+	+	-	-
/	765.9196	20.10	2	+	+	+	-
/	765.9238	20.13	2	+	+	-	-
/	766.7067	13.88	3	+	+	+	+
/	767.4245	8.27	1	+	+	+	-
/	767.7089	14.89	3	+	+	+	-
/	771.3333	10.39	1	+	+	+	+
/	773.3523	12.10		+	+	+	+
/	775.9012	16.04	2	+	+	-	+
/	776.9109	20.13	2	+	+	-	-
/	778.3712	14.88	2	+	+	+	+
/	780.7086	11.86	3	+	+	-	-
/	780.9306	20.10	2	-	+	+	+
/	781.9127	22.43	2	+	+	+	+
/	781.9374	21.52	2	+	+	+	+
/	783.4554	13.03	1	+	+	+	+
/	784.8969	20.13	2	+	+	+	+
/	785.4468	10.98	1	+	+	+	-

/	785.8968	19.81	2	✚	✚	✚	✚
/	786.4556	23.66	1	✚	✚	-	-
/	790.9093	15.95	2	-	✚	✚	-
/	792.8801	18.34	2	✚	✚	✚	✚
/	793.8986	19.40	2	✚	✚	✚	✚
/	795.9306	19.61	2	✚	✚	✚	✚
/	798.9020	16.04	2	✚	✚	-	✚
/	800.3909	11.86	2	✚	✚	✚	-
/	802.9475	21.40	2	✚	✚	-	-
/	804.3942	18.11	2	✚	✚	-	✚
/	804.8810	13.59	2	✚	✚	✚	✚
/	811.4610	12.30	1	✚	✚	✚	✚
/	811.4614	13.13	1	✚	✚	✚	-
/	811.4615	13.20	1	✚	✚	✚	-
/	811.4623	12.27	1	✚	✚	✚	✚
/	811.4634	12.10	1	✚	✚	✚	✚
/	811.7179	18.34	3	✚	✚	✚	✚
/	813.4302	15.75	1	✚	✚	✚	✚
/	816.4244	16.24	2	✚	✚	✚	✚
/	821.3904	21.20	1	✚	✚	✚	✚
/	821.4173	20.49	2	✚	✚	-	-
/	823.5000	15.75	1	✚	✚	✚	-
/	824.4354	19.63	2	✚	✚	✚	✚
/	825.9096	16.49	2	✚	✚	-	-
/	829.4146	19.19	2	✚	✚	-	-
/	834.7436	17.43	3	✚	✚	✚	✚
/	841.4766	20.13	1	✚	✚	✚	✚
/	842.9218	18.95	2	-	✚	✚	-
/	844.4641	23.36	1	✚	✚	✚	-
/	845.4240	22.27	3	✚	✚	✚	✚
/	845.9125	15.33	2	✚	✚	✚	✚
/	849.7429	26.80	3	-	✚	-	✚
/	850.4249	21.02	3	✚	✚	✚	✚
/	852.4311	17.04	1	✚	✚	✚	✚
/	854.4121	14.46	2	✚	✚	✚	✚
/	868.4834	10.62	1	✚	✚	✚	✚
/	868.9328	14.88	2	✚	✚	-	-
/	874.5250	31.78	1	✚	✚	✚	✚
/	874.7801	17.92	3	✚	✚	✚	✚
/	874.9170	14.46	2	✚	✚	-	✚
/	883.4320	12.40	2	✚	✚	-	✚
/	892.5215	14.62	1	✚	✚	✚	✚

/	892.9678	20.00	2	✚	✚	-	-
/	898.5327	16.42	1	✚	✚	✚	✚
/	900.9699	18.89	2	✚	✚	-	-
/	921.4785	16.68	2	✚	✚	-	-
/	926.5504	23.66	1	✚	✚	✚	✚
/	928.4948	12.27	1	✚	✚	✚	✚
/	934.4512	17.75	1	✚	✚	✚	✚
/	937.5318	13.03	1	✚	✚	-	-
/	960.5383	22.63	1	✚	✚	-	-
/	976.5327	21.02	1	✚	✚	-	-
/	1008.4980	12.55	1	✚	✚	✚	-
/	1014.4776	19.81	1	✚	✚	✚	✚
/	1018.5114	19.19	1	✚	✚	✚	✚
/	1021.5555	23.66		✚	✚	✚	✚
/	1027.4908	13.59	1	✚	✚	-	✚
/	1029.4871	19.50	1	✚	✚	✚	✚
/	1078.5911	14.88	1	✚	✚	-	-
/	1088.5613	17.34	1	✚	✚	✚	✚
/	1092.5548	16.04	1	✚	✚	✚	✚
/	1094.5447	27.08	1	✚	✚	✚	✚
/	1105.5997	13.59	1	✚	✚	✚	✚
/	1149.5944	14.46	1	✚	✚	✚	✚
/	1192.6607	15.00	1	✚	✚	✚	✚

Notes: Candidates were firstly screened from BPC of home-made horse-hide gelatin. Subsequently, they further confirmed by comparing EIC of four animal species. "✚" indicates that the marker candidate was detected by UPLC-Q-TOF-MS. "-" indicates that the marker candidate was not detected. These marker candidates showed two/three/four charges and were deduced to be peptides.

Supplementary Table S3

Markers selection for cattle-hide gelatin from peptide fragments based on trypsin digestion followed by LC-Q-TOF-MS analysis.

Cattle marker No.	m/z	RT (min)	Charge	Donkey	Horse	Cattle	Pig
/	189.1288	5.31	1	✚	✚	✚	✚
/	216.6198	8.26	2	✚	✚	✚	✚
/	219.1404	10.98	1	✚	✚	✚	✚
/	223.1141	8.27	1	✚	✚	✚	✚
/	224.1352	31.00	1	✚	✚	✚	✚
/	228.6384	5.31	2	✚	✚	✚	✚
/	229.6280	12.09	2	✚	✚	✚	✚
/	231.1762	10.44	1	✚	✚	✚	✚
/	231.1771	7.77		✚	✚	✚	✚
/	233.1565	5.55	1	✚	✚	✚	✚
/	234.1024	22.27		✚	✚	✚	✚
/	236.6544	9.34	2	✚	✚	✚	✚
/	237.6260	10.08	2	✚	✚	✚	✚
/	237.6447	5.31	2	✚	✚	✚	✚
/	242.1565	10.08	1	✚	✚	✚	✚
/	243.1408	15.75	1	✚	✚	✚	✚
/	245.1925	14.24	1	✚	✚	✚	✚
/	245.1925	15.38	1	✚	✚	✚	✚

/	249.6631	10.08	2	+	+	+	+
/	250.1855	32.78	1	+	+	+	+
/	258.1766	10.10	2	+	+	+	+
/	260.1678	9.58	1	+	+	+	+
/	260.2043	6.27	1	+	+	+	+
/	265.1621	13.32	1	+	+	+	+
/	267.6266	6.27	2	+	+	+	+
/	270.2001	10.08	1	+	+	+	+
/	272.1795	9.40	1	+	+	+	+
/	272.6535	12.27	2	+	+	+	+
/	274.1954	10.08	1	+	+	+	+
/	276.1632	7.57	1	+	+	+	+
/	277.1257	9.74	1	+	+	+	+
/	278.6662	12.50	2	+	+	+	+
/	279.1418	17.53	1	+	+	+	+
/	279.1777	18.20	1	+	+	+	+
/	281.1213	10.08	1	+	+	+	+
/	281.1566	5.59	1	+	+	+	+
/	283.8095	12.27	2	+	+	+	+
/	288.2009	20.49		+	+	+	+
/	288.2116	6.30		+	+	+	+
/	290.1784	6.30		+	+	+	+
/	292.6630	12.27	2	+	+	+	+
/	295.1730	10.98	1	+	+	+	+
/	295.1740	10.08	1	+	+	+	+
/	297.6402	12.50	2	+	+	+	+
/	300.6175	12.78	2	+	+	+	+
/	301.1832	6.30	2	+	+	+	+
/	301.6758	8.27	2	+	+	+	+
/	301.6768	14.24	2	+	+	+	+
/	302.1801	10.08	1	+	+	+	+
/	302.2159	19.22	1	+	+	+	+
/	302.2166	18.21	1	+	+	+	+
/	303.6207	6.30	1	+	+	+	+
/	305.6956	11.52	2	+	+	+	+
/	306.1484	23.08	2	+	+	+	+
/	306.1739	6.30		+	+	+	+
/	309.6738	12.35	2	+	+	+	+
/	312.8329	16.42	3	+	+	+	+
/	314.6831	12.07	2	+	+	+	+
/	314.7013	11.46	2	+	+	+	-
/	316.2312	19.63	1	+	+	+	+

/	317.1910	6.30	3	✚	✚	✚	✚
/	320.1733	14.88	1	✚	✚	✚	✚
/	320.1866	8.27	1	✚	✚	✚	-
/	320.2031	12.78	3	✚	✚	✚	✚
/	321.1797	13.31	2	✚	✚	✚	-
/	321.1817	5.59	1	✚	✚	✚	✚
/	322.6805	10.06	2	✚	✚	✚	✚
/	322.8205	12.27	3	✚	✚	✚	✚
/	323.1587	6.30		✚	✚	✚	✚
/	323.4940	6.30		✚	✚	✚	✚
/	324.1811	5.59	2	✚	✚	✚	✚
/	327.8098	12.27	3	✚	✚	✚	✚
/	329.1909	6.30	2	✚	✚	✚	✚
/	329.2007	5.31	1	✚	✚	✚	✚
/	329.2282	15.00	2	✚	✚	✚	✚
/	330.1635	19.95	1	✚	✚	✚	✚
/	330.2011	12.78	2	✚	✚	✚	✚
/	332.2266	14.24	1	✚	✚	✚	✚
/	332.2273	18.89	1	✚	✚	✚	✚
/	335.1611	21.76	3	✚	✚	✚	✚
/	339.1935	10.87	2	✚	✚	✚	✚
/	339.6729	11.52	2	✚	✚	✚	✚
/	343.2051	12.27		✚	✚	✚	✚
/	344.2632	20.16	1	✚	✚	✚	✚
/	345.1529	9.63	1	✚	✚	✚	✚
/	345.2333	5.31	1	✚	✚	✚	✚
/	347.2014	7.77	1	✚	✚	✚	✚
/	347.7072	11.46	2	✚	✚	✚	✚
/	350.1806	9.63	1	✚	✚	✚	✚
/	350.6348	14.08	2	✚	✚	✚	✚
/	353.4912	14.88	3	✚	✚	✚	✚
/	359.2392	10.08	1	✚	✚	✚	✚
/	359.6404	14.08	2	✚	✚	✚	✚
/	365.8721	8.27		✚	✚	✚	-
/	366.2176	17.35	2	✚	✚	✚	✚
/	369.2061	13.59	3	✚	✚	✚	✚
/	369.7033	6.30	2	✚	✚	✚	✚
/	371.2385	10.98	1	✚	✚	✚	✚
/	371.7236	16.41	2	✚	✚	✚	✚
/	372.2159	10.36	2	✚	✚	✚	✚
/	372.6956	5.59	2	✚	✚	✚	✚
/	373.2899	6.30	1	✚	✚	✚	✚

/	374.2389	18.89	1	✚	✚	✚	✚
/	374.7011	12.41	2	✚	✚	✚	✚
/	374.8536	14.46	3	✚	✚	✚	✚
/	375.2342	7.77	1	✚	✚	✚	✚
/	377.1926	13.59	1	✚	✚	✚	✚
/	377.1953	11.46	1	✚	✚	✚	✚
/	378.6137	14.08	2	✚	✚	✚	✚
/	379.2181	24.94	2	✚	✚	✚	✚
/	379.7035	10.98	2	✚	✚	✚	✚
/	379.8415	14.46	3	✚	✚	✚	✚
/	381.8550	13.59	3	✚	✚	✚	✚
/	382.5030	14.46	3	✚	✚	✚	✚
/	383.7419	15.00	2	✚	✚	✚	-
/	384.2149	8.64	2	✚	✚	✚	-
/	385.2063	8.27	2	✚	✚	✚	✚
/	385.2297	10.08	1	✚	✚	✚	✚
/	385.2661	9.40	1	✚	✚	✚	✚
/	386.8436	13.59	3	✚	✚	✚	✚
/	387.8462	19.63	3	✚	✚	✚	✚
/	388.2293	6.30		✚	✚	✚	✚
/	388.2296	12.27		✚	✚	✚	✚
/	389.2488	11.01	1	✚	✚	✚	✚
/	390.2222	10.60	2	✚	✚	✚	✚
/	391.1910	5.31	1	✚	✚	✚	✚
/	391.2092	18.32	1	✚	✚	✚	✚
/	392.2315	13.05	2	✚	✚	✚	✚
/	393.2091	9.40	2	✚	✚	✚	✚
/	393.2267	11.01	2	✚	✚	✚	-
/	395.6893	8.94	2	✚	✚	✚	✚
/	396.2707	14.62	1	✚	✚	✚	✚
/	396.6962	20.13	4	✚	✚	✚	-
/	398.2194	10.08		✚	✚	✚	-
/	398.2272	15.00	3	✚	✚	✚	✚
/	398.2504	14.18	1	✚	✚	✚	✚
/	399.2711	17.34	1	✚	✚	✚	✚
/	400.2297	9.40	1	✚	✚	✚	✚
/	400.2298	12.78	2	✚	✚	✚	✚
/	402.2567	10.08	1	✚	✚	✚	✚
/	402.5164	22.63	3	✚	✚	✚	✚
/	403.2657	15.00	1	✚	✚	✚	✚
/	404.2236	9.40	1	✚	✚	✚	✚
/	404.4604	19.63	4	✚	✚	✚	✚

/	405.2186	9.63	2	✚	✚	✚	✚
/	406.2352	13.25	2	✚	✚	✚	-
/	406.2358	12.21	2	✚	✚	✚	✚
/	407.2195	15.76	2	✚	✚	✚	✚
/	407.9573	19.63	4	✚	✚	✚	✚
/	410.8747	15.00	3	✚	✚	✚	✚
/	411.2322	10.98	1	✚	✚	✚	✚
/	411.6992	19.63	4	✚	✚	✚	✚
/	412.2536	16.04	2	✚	✚	✚	✚
/	412.2540	15.75	2	✚	✚	✚	✚
/	413.2410	14.18	2	✚	✚	✚	✚
/	413.2866	21.76	1	✚	✚	✚	✚
/	414.2413	14.88	3	✚	✚	✚	✚
/	414.2457	12.27	1	✚	✚	✚	✚
/	415.2447	22.63	1	✚	✚	✚	✚
/	415.8626	15.00	3	✚	✚	✚	✚
/	418.2195	17.95	1	✚	✚	✚	✚
/	418.7339	10.36	2	✚	✚	✚	✚
/	420.1979	13.01	1	✚	✚	✚	✚
/	421.2412	12.45	2	✚	✚	✚	✚
/	421.2432	20.13	2	✚	✚	✚	✚
/	421.2612	15.00	2	✚	✚	✚	✚
/	421.7331	9.63	2	✚	✚	✚	✚
/	422.2127	5.31	1	✚	✚	✚	✚
/	426.7299	8.94	2	✚	✚	✚	✚
/	429.2457	22.30	1	✚	✚	✚	✚
/	429.2826	21.01	1	✚	✚	✚	✚
/	430.2411	12.78	1	✚	✚	✚	✚
/	430.2775	20.63	1	✚	✚	✚	✚
/	431.2253	13.59	2	✚	✚	✚	✚
/	431.2478	6.30	1	✚	✚	✚	✚
/	431.5276	21.95	3	✚	✚	✚	✚
/	432.2315	8.27	1	✚	✚	✚	✚
/	434.2619	17.34	2	✚	✚	✚	✚
/	434.7471	10.81	2	✚	✚	✚	✚
/	437.7673	31.77	2	✚	✚	✚	✚
/	441.2521	20.49	2	✚	✚	✚	✚
/	441.7730	18.54	2	✚	✚	✚	✚
/	442.7441	12.27	2	✚	✚	✚	✚
/	443.2353	6.30	1	✚	✚	✚	✚
/	444.2303	10.08	2	✚	✚	✚	✚
/	444.2936	20.49	1	✚	✚	✚	✚

/	445.2527	7.80	1	✚	✚	✚	✚
/	446.2717	8.27	1	✚	✚	✚	✚
/	446.2722	16.69	1	✚	✚	✚	✚
/	446.7472	11.52	2	✚	✚	✚	✚
/	446.7659	14.62	2	✚	✚	✚	✚
/	447.2085	13.59	1	✚	✚	✚	✚
/	447.2105	6.30	1	✚	✚	✚	✚
/	447.2542	14.60	1	✚	✚	✚	✚
/	448.2467	10.36	2	✚	✚	✚	✚
/	449.7704	16.48	2	✚	✚	✚	✚
/	451.2280	22.27	1	✚	✚	✚	✚
/	453.2203	12.27		✚	✚	✚	✚
/	454.7644	13.80	2	✚	✚	✚	✚
/	455.2042	19.61	1	✚	✚	✚	✚
/	456.2434	8.94	2	✚	✚	✚	✚
/	456.2456	13.15	2	✚	✚	✚	✚
/	456.2925	23.26	1	✚	✚	✚	✚
/	457.2030	14.88	2	✚	✚	✚	✚
/	457.7509	12.27	2	✚	✚	✚	✚
/	458.2476	12.10	2	✚	✚	✚	✚
/	459.1892	12.27	2	✚	✚	✚	✚
/	459.2278	19.63	3	✚	✚	✚	✚
/	459.2678	10.98	1	✚	✚	✚	✚
/	459.2685	5.59	1	✚	✚	✚	✚
/	459.2935	21.96	1	✚	✚	✚	✚
/	462.2302	6.30	1	✚	✚	✚	✚
/	462.2472	19.19	1	✚	✚	✚	✚
/	464.2263	13.90	1	✚	✚	✚	✚
/	464.7522	12.26	2	✚	✚	✚	✚
/	465.2464	14.88	1	✚	✚	✚	✚
/	467.7294	17.95	2	✚	✚	✚	✚
/	467.7517	11.01	2	✚	✚	✚	✚
/	468.2369	11.52	1	✚	✚	✚	✚
/	471.7388	12.84	2	✚	✚	✚	✚
/	472.2880	13.31	1	✚	✚	✚	✚
/	472.2893	21.02	1	✚	✚	✚	✚
/	472.2992	9.40	1	✚	✚	✚	✚
/	472.5596	18.89	3	✚	✚	✚	✚
/	473.2079	18.21	2	✚	✚	✚	✚
/	473.2346	5.59		✚	✚	✚	-
/	474.2428	11.52	1	✚	✚	✚	✚
/	474.2429	10.08	1	✚	✚	✚	✚

/	474.2679	12.27	2	✚	✚	✚	✚
/	474.2682	12.27	1	✚	✚	✚	✚
/	474.2799	5.31	1	✚	✚	✚	✚
/	475.7502	10.57	2	✚	✚	✚	✚
/	476.2273	18.89	1	✚	✚	✚	✚
/	476.2476	12.35	2	✚	✚	✚	✚
/	478.7565	13.59	2	✚	✚	✚	✚
/	479.2622	17.50	1	✚	✚	✚	✚
/	479.7979	12.78	2	✚	✚	✚	✚
/	480.2565	21.76	1	✚	✚	✚	✚
/	484.2314	6.30	2	✚	✚	✚	✚
/	486.3042	14.60	1	✚	✚	✚	✚
/	486.3052	14.62	1	✚	✚	✚	✚
/	490.2600	5.59		✚	✚	✚	✚
/	493.2424	19.19	1	✚	✚	✚	✚
/	498.7538	9.63	2	✚	✚	✚	✚
/	499.2625	7.80	1	✚	✚	✚	✚
/	499.7645	21.01	2	✚	✚	✚	✚
/	501.2429	6.30	1	✚	✚	✚	✚
/	501.7630	24.02	2	✚	✚	✚	✚
/	504.7541	12.50	2	✚	✚	✚	-
/	507.7421	19.80	2	✚	✚	✚	✚
/	508.2906	15.50	1	✚	✚	✚	✚
/	509.7630	19.19	2	✚	✚	✚	✚
/	510.2391	16.77	2	✚	✚	✚	✚
/	511.2845	23.66	2	✚	✚	✚	✚
/	511.7312	19.81	2	✚	✚	✚	✚
/	512.2539	16.68	2	✚	✚	✚	✚
/	512.5736	19.63	3	✚	✚	✚	✚
/	512.7552	24.02	2	✚	✚	✚	✚
/	513.7482	20.49	2	✚	✚	✚	✚
/	515.2510	19.50	2	✚	✚	✚	✚
/	515.3429	10.06	1	✚	✚	✚	✚
/	518.7344	19.81	2	✚	✚	✚	✚
/	520.7371	24.02	2	✚	✚	✚	✚
/	520.9586	20.16	3	-	✚	✚	-
/	524.7756	11.14	2	-	✚	✚	-
/	524.8132	17.94	2	✚	✚	✚	-
/	525.7364	25.65	2	-	-	✚	✚
/	526.2750	5.31	1	✚	✚	✚	✚
/	526.7177	19.81	2	✚	✚	✚	✚
/	527.2957	12.10	1	✚	✚	✚	✚

/	528.9272	20.10	3	✚	✚	✚	-
/	529.6020	19.40	3	✚	✚	✚	✚
/	530.7887	18.89	2	✚	✚	✚	✚
/	530.9570	19.63	3	✚	✚	✚	✚
/	531.2907	12.27	1	✚	✚	✚	✚
/	531.7855	12.78	2	✚	✚	✚	✚
/	534.1999	19.81	2	✚	✚	✚	✚
/	534.2432	6.30	1	✚	✚	✚	✚
/	535.2696	14.08	1	✚	✚	✚	✚
/	535.8036	17.75	2	✚	✚	✚	✚
/	536.7798	12.35	2	✚	✚	✚	-
/	538.2274	12.65	2	✚	✚	✚	✚
/	538.2836	19.63	3	✚	✚	✚	✚
/	538.7866	17.27	2	✚	✚	✚	✚
/	539.7781	28.50	2	✚	✚	✚	✚
/	542.7990	14.46	2	✚	✚	✚	✚
/	543.6046	19.63	3	✚	✚	✚	✚
/	544.2973	12.40	1	✚	✚	✚	✚
/	544.3862	26.81	1	✚	✚	✚	✚
/	544.7864	16.68	2	✚	✚	✚	✚
/	544.7866	17.43	2	✚	✚	✚	✚
/	545.3081	14.46	2	✚	✚	✚	✚
/	546.7834	16.04	2	✚	✚	✚	✚
/	547.7753	27.08	2	✚	✚	✚	✚
/	548.9296	19.63	3	✚	✚	✚	✚
/	550.7959	14.24	2	✚	✚	✚	✚
/	552.6142	12.58	3	✚	✚	✚	✚
/	553.3052	13.59	2	✚	✚	✚	✚
/	553.7987	13.56	2	✚	✚	✚	✚
/	556.3212	12.55	1	✚	✚	✚	✚
/	556.7927	17.20	2	✚	✚	✚	✚
/	558.3128	5.59		✚	✚	✚	✚
/	558.7653	27.09	2	✚	✚	✚	✚
/	560.3174	13.31	1	✚	✚	✚	✚
/	561.3171	24.91	1	✚	✚	✚	✚
/	562.2794	12.78	1	✚	✚	✚	✚
/	563.8012	17.94	2	✚	✚	✚	✚
/	564.2834	15.75	1	✚	✚	✚	✚
/	564.2942	13.60	2	✚	✚	✚	✚
/	568.2868	6.30	1	✚	✚	✚	✚
/	569.6130	14.46	3	✚	✚	✚	✚
/	569.7932	10.06	2	✚	✚	✚	✚

/	570.3125	12.10	1	✚	✚	✚	✚
/	571.2687	13.00	1	-	✚	✚	✚
/	571.2819	12.27	1	✚	✚	✚	✚
/	571.3244	19.81	1	✚	✚	✚	-
/	572.2779	13.60	2	✚	✚	✚	✚
/	572.3803	25.69	1	✚	✚	✚	✚
/	573.2504	14.46	2	✚	✚	✚	✚
/	573.2644	5.31	1	✚	✚	✚	✚
/	573.3398	23.14	1	✚	✚	✚	✚
/	574.2337	27.09	2	✚	✚	✚	✚
/	575.3018	14.46	2	✚	✚	✚	✚
/	578.2752	5.31	1	✚	✚	✚	✚
/	578.3000	12.27	2	✚	✚	✚	✚
/	578.3056	12.53	1	✚	✚	✚	✚
/	579.7647	13.59	2	✚	✚	✚	✚
/	581.2635	19.63	2	✚	✚	✚	✚
/	581.3193	11.52	1	✚	✚	✚	✚
/	581.8293	17.50	2	✚	✚	✚	✚
/	583.8135	12.10	2	✚	✚	✚	✚
/	584.3129	6.30		✚	✚	✚	✚
/	586.3359	16.42	1	✚	✚	✚	✚
/	587.1737	14.08	1	✚	✚	✚	✚
/	587.3193	16.26	1	✚	✚	✚	-
/	588.8368	16.04	2	✚	✚	✚	✚
/	589.3338	15.44	1	✚	✚	✚	✚
/	591.8236	21.95	2	✚	✚	✚	-
/	594.2725	14.46	2	✚	✚	✚	✚
/	596.3298	12.10	1	✚	✚	✚	✚
/	596.8323	14.97	2	✚	✚	✚	✚
**1**	596.8454	18.90	2	-	-	✚	-
/	599.3923	22.27	1	✚	✚	✚	✚
/	600.2304	12.78		✚	✚	✚	✚
/	600.3867	20.13	1	✚	✚	✚	✚
/	601.3122	18.39	2	✚	✚	✚	✚
/	602.2936	14.24	1	✚	✚	✚	✚
/	602.3390	8.27	1	✚	✚	✚	✚
/	603.2689	22.63	2	✚	✚	✚	✚
/	604.2905	12.35	1	✚	✚	✚	✚
/	604.3075	9.40	1	✚	✚	✚	✚
**2**	604.8556	17.84	2	-	-	✚	-
/	607.2546	15.50	1	✚	✚	✚	✚
/	607.2905	19.63	1	✚	✚	✚	✚

/	607.8255	14.80	2	✚	✚	✚	✚
/	608.3549	16.74	1	✚	✚	✚	✚
/	609.8244	17.75	2	✚	✚	✚	✚
/	618.3392	12.35	1	✚	✚	✚	✚
/	621.3747	21.95	1	✚	✚	✚	✚
/	621.9774	17.10	3	✚	✚	✚	-
/	624.3507	15.75	1	✚	✚	✚	✚
/	628.3564	12.10	2	✚	✚	✚	✚
/	629.8132	12.78	2	✚	✚	✚	✚
/	641.3310	23.36	2	✚	✚	✚	✚
/	643.3687	16.42	1	✚	✚	✚	✚
/	644.3510	10.06	1	✚	✚	✚	✚
/	644.8290	16.75	2	✚	✚	✚	-
/	646.7862	21.95	2	✚	✚	✚	✚
/	647.3307	17.75	1	✚	✚	✚	✚
/	648.3511	20.10	2	✚	✚	✚	✚
/	648.8497	23.36	2	✚	✚	✚	✚
/	649.3000	19.82	1	✚	✚	✚	✚
/	649.3232	22.63	2	✚	✚	✚	✚
/	654.3328	17.94	2	✚	✚	✚	✚
/	655.3930	13.03	1	✚	✚	✚	✚
/	655.8360	14.50	2	✚	✚	✚	-
/	656.8466	21.95	2	✚	✚	✚	✚
/	660.3497	15.00	2	✚	✚	✚	✚
/	662.3516	17.34	2	✚	✚	✚	✚
/	664.8443	20.70	2	✚	✚	✚	✚
/	666.8469	13.20	2	✚	✚	✚	✚
/	670.3497	15.50	2	✚	✚	✚	✚
/	670.4318	23.36	1	✚	✚	✚	✚
/	672.3542	18.90	2	✚	✚	✚	✚
/	672.8430	20.00	2	✚	✚	✚	✚
/	677.3781	10.98	1	✚	✚	✚	✚
/	683.8020	20.49	2	✚	✚	✚	✚
/	688.4038	20.13	1	✚	✚	✚	✚
/	690.3999	25.69	1	-	✚	✚	-
/	694.4061	11.52	1	✚	✚	✚	✚
/	696.8466	13.03	2	✚	✚	✚	-
/	703.3402	14.46	2	✚	✚	✚	✚
/	705.8646	21.52	2	✚	✚	✚	-
/	708.3562	18.89	2	✚	✚	✚	✚
/	708.6879	14.08	3	✚	✚	✚	✚
/	710.8729	15.35	2	✚	✚	✚	✚

/	713.0401	19.05	3	✚	✚	✚	-
/	718.2710	14.08	1	✚	✚	✚	✚
/	718.7115	18.11	3	✚	✚	✚	-
/	723.3107	13.49	1	✚	✚	✚	✚
/	723.7094	17.26	3	✚	✚	✚	-
/	724.0438	17.34	3	✚	✚	✚	-
/	726.3114	20.13	2	✚	✚	✚	✚
/	726.3605	11.86	1	✚	✚	✚	✚
/	730.3692	16.42	2	✚	✚	✚	✚
/	732.3417	17.04	1	✚	✚	✚	✚
/	745.3828	5.59		✚	✚	✚	✚
/	745.8669	15.33	2	-	✚	✚	-
/	748.3914	12.40	1	✚	✚	✚	✚
/	749.0318	14.89	3	✚	✚	✚	✚
/	753.8639	14.89	2	✚	✚	✚	✚
/	755.7079	15.33	3	✚	✚	✚	✚
/	757.4285	24.97	1	✚	✚	✚	-
/	761.0394	14.46	3	✚	✚	✚	✚
/	765.9196	20.10	2	✚	✚	✚	-
/	766.7067	13.88	3	✚	✚	✚	✚
**3**	766.8957	18.34	2	-	-	✚	-
/	767.4245	8.27	1	✚	✚	✚	-
/	767.7089	14.89	3	✚	✚	✚	-
/	771.3333	10.39	1	✚	✚	✚	✚
/	773.3523	12.10		✚	✚	✚	✚
/	778.3712	14.88	2	✚	✚	✚	✚
/	780.9306	20.10	2	-	✚	✚	✚
/	781.9127	22.43	2	✚	✚	✚	✚
/	781.9374	21.52	2	✚	✚	✚	✚
/	783.4554	13.03	1	✚	✚	✚	✚
/	784.8969	20.13	2	✚	✚	✚	✚
/	785.4468	10.98	1	✚	✚	✚	-
/	785.8968	19.81	2	✚	✚	✚	✚
/	790.9093	15.95	2	-	✚	✚	-
/	792.8801	18.34	2	✚	✚	✚	✚
/	793.8986	19.40	2	✚	✚	✚	✚
/	795.9306	19.61	2	✚	✚	✚	✚
/	800.3909	11.86	2	✚	✚	✚	-
/	804.8810	13.59	2	✚	✚	✚	✚
/	811.4610	12.30	1	✚	✚	✚	✚
/	811.4614	13.13	1	✚	✚	✚	-
/	811.4615	13.20	1	✚	✚	✚	-

/	811.4623	12.27	1	✚	✚	✚	✚
/	811.4634	12.10	1	✚	✚	✚	✚
/	811.7179	18.34	3	✚	✚	✚	✚
/	813.4302	15.75	1	✚	✚	✚	✚
/	816.4244	16.24	2	✚	✚	✚	✚
/	821.3904	21.20	1	✚	✚	✚	✚
/	823.5000	15.75	1	✚	✚	✚	-
/	824.4354	19.63	2	✚	✚	✚	✚
/	834.7436	17.43	3	✚	✚	✚	✚
/	841.4766	20.13	1	✚	✚	✚	✚
/	842.9218	18.95	2	-	✚	✚	-
/	844.4641	23.36	1	✚	✚	✚	-
/	845.4240	22.27	3	✚	✚	✚	✚
/	845.9125	15.33	2	✚	✚	✚	✚
/	850.4249	21.02	3	✚	✚	✚	✚
/	852.4311	17.04	1	✚	✚	✚	✚
/	854.4121	14.46	2	✚	✚	✚	✚
/	868.4834	10.62	1	✚	✚	✚	✚
/	874.5250	31.78	1	✚	✚	✚	✚
/	874.7801	17.92	3	✚	✚	✚	✚
/	892.5215	14.62	1	✚	✚	✚	✚
/	898.5327	16.42	1	✚	✚	✚	✚
/	926.5504	23.66	1	✚	✚	✚	✚
/	928.4948	12.27	1	✚	✚	✚	✚
/	934.4512	17.75	1	✚	✚	✚	✚
/	1008.4980	12.55	1	✚	✚	✚	-
/	1014.4776	19.81	1	✚	✚	✚	✚
/	1018.5114	19.19	1	✚	✚	✚	✚
/	1021.5555	23.66		✚	✚	✚	✚
/	1029.4871	19.50	1	✚	✚	✚	✚
/	1088.5613	17.34	1	✚	✚	✚	✚
/	1092.5548	16.04	1	✚	✚	✚	✚
/	1094.5447	27.08	1	✚	✚	✚	✚
/	1105.5997	13.59	1	✚	✚	✚	✚
/	1149.5944	14.46	1	✚	✚	✚	✚
/	1192.6607	15.00	1	✚	✚	✚	✚

Notes: Candidates were firstly screened from BPC of standard substance of cattle-hide gelatin. Subsequently, they further confirmed by comparing EIC of four animal species. "✚" indicates that the marker candidate was detected by UPLC-Q-TOF-MS. "-" indicates that the marker candidate was not detected. These marker candidates showed two/three/four charges and were deduced to be peptides.

Supplementary Table S4

Markers selection for pig-hide gelatin from peptide fragments based on trypsin digestion followed by LC-Q-TOF-MS analysis.

Pig marker No.	m/z	RT (min)	Charge	Donkey	Horse	Cattle	Pig
/	189.1288	5.31	1	✚	✚	✚	✚
/	216.6198	8.26	2	✚	✚	✚	✚
/	219.1404	10.98	1	✚	✚	✚	✚
/	223.1141	8.27	1	✚	✚	✚	✚
/	224.1352	31.00	1	✚	✚	✚	✚
/	228.6384	5.31	2	✚	✚	✚	✚
/	229.6280	12.09	2	✚	✚	✚	✚
/	231.1762	10.44	1	✚	✚	✚	✚
/	231.1771	7.77		✚	✚	✚	✚
/	233.1565	5.55	1	✚	✚	✚	✚
/	234.1024	22.27		✚	✚	✚	✚
/	236.6544	9.34	2	✚	✚	✚	✚
/	237.6260	10.08	2	✚	✚	✚	✚
/	237.6447	5.31	2	✚	✚	✚	✚
/	242.1565	10.08	1	✚	✚	✚	✚
/	243.1408	15.75	1	✚	✚	✚	✚
/	245.1925	14.24	1	✚	✚	✚	✚
/	245.1925	15.38	1	✚	✚	✚	✚
/	249.6631	10.08	2	✚	✚	✚	✚
/	250.1855	32.78	1	✚	✚	✚	✚
/	258.1766	10.10	2	✚	✚	✚	✚
/	260.1678	9.58	1	✚	✚	✚	✚
/	260.2043	6.27	1	✚	✚	✚	✚
/	265.1621	13.32	1	✚	✚	✚	✚
/	267.6266	6.27	2	✚	✚	✚	✚
/	270.2001	10.08	1	✚	✚	✚	✚
/	272.1795	9.40	1	✚	✚	✚	✚
/	272.6535	12.27	2	✚	✚	✚	✚
/	274.1954	10.08	1	✚	✚	✚	✚
/	276.1632	7.57	1	✚	✚	✚	✚
/	277.1257	9.74	1	✚	✚	✚	✚
/	278.6662	12.50	2	✚	✚	✚	✚
/	279.1418	17.53	1	✚	✚	✚	✚
/	279.1777	18.20	1	✚	✚	✚	✚
/	281.1213	10.08	1	✚	✚	✚	✚
/	281.1566	5.59	1	✚	✚	✚	✚
/	283.8095	12.27	2	✚	✚	✚	✚
/	288.2009	20.49		✚	✚	✚	✚
/	288.2116	6.30		✚	✚	✚	✚

/	290.1784	6.30		✚	✚	✚	✚
/	292.6630	12.27	2	✚	✚	✚	✚
/	295.1730	10.98	1	✚	✚	✚	✚
/	295.1740	10.08	1	✚	✚	✚	✚
/	297.6402	12.50	2	✚	✚	✚	✚
/	300.6175	12.78	2	✚	✚	✚	✚
/	301.1832	6.30	2	✚	✚	✚	✚
/	301.6758	8.27	2	✚	✚	✚	✚
/	301.6768	14.24	2	✚	✚	✚	✚
/	302.1801	10.08	1	✚	✚	✚	✚
/	302.2159	19.22	1	✚	✚	✚	✚
/	302.2166	18.21	1	✚	✚	✚	✚
/	303.6207	6.30	1	✚	✚	✚	✚
/	305.6956	11.52	2	✚	✚	✚	✚
/	306.1484	23.08	2	✚	✚	✚	✚
/	306.1739	6.30		✚	✚	✚	✚
/	309.6738	12.35	2	✚	✚	✚	✚
/	312.8329	16.42	3	✚	✚	✚	✚
/	314.6831	12.07	2	✚	✚	✚	✚
/	316.2312	19.63	1	✚	✚	✚	✚
/	317.1910	6.30	3	✚	✚	✚	✚
/	320.1733	14.88	1	✚	✚	✚	✚
/	320.2031	12.78	3	✚	✚	✚	✚
/	321.1817	5.59	1	✚	✚	✚	✚
/	322.6805	10.06	2	✚	✚	✚	✚
/	322.8205	12.27	3	✚	✚	✚	✚
/	323.1587	6.30		✚	✚	✚	✚
/	323.4940	6.30		✚	✚	✚	✚
/	324.1811	5.59	2	✚	✚	✚	✚
/	327.8098	12.27	3	✚	✚	✚	✚
/	329.1909	6.30	2	✚	✚	✚	✚
/	329.2007	5.31	1	✚	✚	✚	✚
/	329.2282	15.00	2	✚	✚	✚	✚
/	330.1635	19.95	1	✚	✚	✚	✚
/	330.2011	12.78	2	✚	✚	✚	✚
/	332.2266	14.24	1	✚	✚	✚	✚
/	332.2273	18.89	1	✚	✚	✚	✚
/	335.1611	21.76	3	✚	✚	✚	✚
/	339.1935	10.87	2	✚	✚	✚	✚
/	339.6729	11.52	2	✚	✚	✚	✚
/	343.2051	12.27		✚	✚	✚	✚
/	344.2632	20.16	1	✚	✚	✚	✚

/	345.1529	9.63	1	+	+	+	+
/	345.2333	5.31	1	+	+	+	+
/	347.2014	7.77	1	+	+	+	+
/	347.7072	11.46	2	+	+	+	+
/	350.1806	9.63	1	+	+	+	+
/	350.6348	14.08	2	+	+	+	+
/	353.4912	14.88	3	+	+	+	+
/	359.2392	10.08	1	+	+	+	+
/	359.6404	14.08	2	+	+	+	+
/	366.2176	17.35	2	+	+	+	+
/	369.2061	13.59	3	+	+	+	+
/	369.7033	6.30	2	+	+	+	+
/	371.2385	10.98	1	+	+	+	+
/	371.7236	16.41	2	+	+	+	+
/	372.2159	10.36	2	+	+	+	+
/	372.6956	5.59	2	+	+	+	+
/	373.2899	6.30	1	+	+	+	+
/	374.2389	18.89	1	+	+	+	+
/	374.7011	12.41	2	+	+	+	+
/	374.8536	14.46	3	+	+	+	+
/	375.2342	7.77	1	+	+	+	+
/	377.1926	13.59	1	+	+	+	+
/	377.1953	11.46	1	+	+	+	+
/	378.6137	14.08	2	+	+	+	+
/	379.2181	24.94	2	+	+	+	+
/	379.7035	10.98	2	+	+	+	+
/	379.8415	14.46	3	+	+	+	+
/	381.8550	13.59	3	+	+	+	+
/	382.5030	14.46	3	+	+	+	+
/	385.2063	8.27	2	+	+	+	+
/	385.2297	10.08	1	+	+	+	+
/	385.2661	9.40	1	+	+	+	+
/	386.8436	13.59	3	+	+	+	+
/	387.8462	19.63	3	+	+	+	+
/	388.2293	6.30		+	+	+	+
/	388.2296	12.27		+	+	+	+
/	389.2488	11.01	1	+	+	+	+
/	390.2222	10.60	2	+	+	+	+
/	391.1910	5.31	1	+	+	+	+
/	391.2092	18.32	1	+	+	+	+
/	392.2315	13.05	2	+	+	+	+
/	393.2091	9.40	2	+	+	+	+

/	395.6893	8.94	2	✚	✚	✚	✚
/	396.2707	14.62	1	✚	✚	✚	✚
/	398.1658	6.30		✚	✚	-	✚
/	398.2272	15.00	3	✚	✚	✚	✚
/	398.2504	14.18	1	✚	✚	✚	✚
/	399.2711	17.34	1	✚	✚	✚	✚
/	400.2297	9.40	1	✚	✚	✚	✚
/	400.2298	12.78	2	✚	✚	✚	✚
/	402.2567	10.08	1	✚	✚	✚	✚
/	402.5164	22.63	3	✚	✚	✚	✚
/	403.2657	15.00	1	✚	✚	✚	✚
/	404.2236	9.40	1	✚	✚	✚	✚
/	404.4604	19.63	4	✚	✚	✚	✚
/	405.2186	9.63	2	✚	✚	✚	✚
/	406.2358	12.21	2	✚	✚	✚	✚
/	407.2195	15.76	2	✚	✚	✚	✚
/	407.9573	19.63	4	✚	✚	✚	✚
/	410.8747	15.00	3	✚	✚	✚	✚
/	411.2322	10.98	1	✚	✚	✚	✚
/	411.6992	19.63	4	✚	✚	✚	✚
/	412.2536	16.04	2	✚	✚	✚	✚
/	412.2540	15.75	2	✚	✚	✚	✚
/	413.2410	14.18	2	✚	✚	✚	✚
/	413.2866	21.76	1	✚	✚	✚	✚
/	414.2413	14.88	3	✚	✚	✚	✚
/	414.2457	12.27	1	✚	✚	✚	✚
/	415.2447	22.63	1	✚	✚	✚	✚
/	415.8626	15.00	3	✚	✚	✚	✚
/	418.2195	17.95	1	✚	✚	✚	✚
/	418.7339	10.36	2	✚	✚	✚	✚
**1**	419.2446	14.04	2	-	-	-	✚
/	420.1979	13.01	1	✚	✚	✚	✚
/	421.2412	12.45	2	✚	✚	✚	✚
/	421.2432	20.13	2	✚	✚	✚	✚
/	421.2612	15.00	2	✚	✚	✚	✚
/	421.7331	9.63	2	✚	✚	✚	✚
/	422.2127	5.31	1	✚	✚	✚	✚
/	426.7299	8.94	2	✚	✚	✚	✚
/	429.2457	22.30	1	✚	✚	✚	✚
/	429.2826	21.01	1	✚	✚	✚	✚
/	430.2411	12.78	1	✚	✚	✚	✚
/	430.2775	20.63	1	✚	✚	✚	✚

/	431.2253	13.59	2	✚	✚	✚	✚
/	431.2478	6.30	1	✚	✚	✚	✚
/	431.5276	21.95	3	✚	✚	✚	✚
/	432.2315	8.27	1	✚	✚	✚	✚
/	434.2619	17.34	2	✚	✚	✚	✚
/	434.7471	10.81	2	✚	✚	✚	✚
/	437.7673	31.77	2	✚	✚	✚	✚
/	441.2521	20.49	2	✚	✚	✚	✚
/	441.7730	18.54	2	✚	✚	✚	✚
/	442.7441	12.27	2	✚	✚	✚	✚
/	443.2353	6.30	1	✚	✚	✚	✚
/	444.2303	10.08	2	✚	✚	✚	✚
/	444.2936	20.49	1	✚	✚	✚	✚
/	445.2527	7.80	1	✚	✚	✚	✚
/	446.2717	8.27	1	✚	✚	✚	✚
/	446.2722	16.69	1	✚	✚	✚	✚
/	446.7472	11.52	2	✚	✚	✚	✚
/	446.7659	14.62	2	✚	✚	✚	✚
/	447.2085	13.59	1	✚	✚	✚	✚
/	447.2105	6.30	1	✚	✚	✚	✚
/	447.2542	14.60	1	✚	✚	✚	✚
/	448.2467	10.36	2	✚	✚	✚	✚
/	449.7704	16.48	2	✚	✚	✚	✚
/	451.2280	22.27	1	✚	✚	✚	✚
/	453.2203	12.27		✚	✚	✚	✚
/	454.7644	13.80	2	✚	✚	✚	✚
/	455.2042	19.61	1	✚	✚	✚	✚
/	456.2434	8.94	2	✚	✚	✚	✚
/	456.2456	13.15	2	✚	✚	✚	✚
/	456.2925	23.26	1	✚	✚	✚	✚
/	457.2030	14.88	2	✚	✚	✚	✚
/	457.7509	12.27	2	✚	✚	✚	✚
/	458.2476	12.10	2	✚	✚	✚	✚
/	459.1892	12.27	2	✚	✚	✚	✚
/	459.2278	19.63	3	✚	✚	✚	✚
/	459.2678	10.98	1	✚	✚	✚	✚
/	459.2685	5.59	1	✚	✚	✚	✚
/	459.2935	21.96	1	✚	✚	✚	✚
/	462.2302	6.30	1	✚	✚	✚	✚
/	462.2472	19.19	1	✚	✚	✚	✚
/	464.2263	13.90	1	✚	✚	✚	✚
/	464.7522	12.26	2	✚	✚	✚	✚

/	465.2464	14.88	1	+	+	+	+
/	467.7294	17.95	2	+	+	+	+
/	467.7517	11.01	2	+	+	+	+
/	468.2369	11.52	1	+	+	+	+
/	471.7388	12.84	2	+	+	+	+
/	472.2880	13.31	1	+	+	+	+
/	472.2893	21.02	1	+	+	+	+
/	472.2992	9.40	1	+	+	+	+
/	472.5596	18.89	3	+	+	+	+
/	473.2079	18.21	2	+	+	+	+
/	474.2428	11.52	1	+	+	+	+
/	474.2429	10.08	1	+	+	+	+
/	474.2679	12.27	2	+	+	+	+
/	474.2682	12.27	1	+	+	+	+
/	474.2799	5.31	1	+	+	+	+
/	475.7502	10.57	2	+	+	+	+
/	476.2273	18.89	1	+	+	+	+
/	476.2476	12.35	2	+	+	+	+
/	478.2575	16.50	2	+	+	-	+
/	478.7565	13.59	2	+	+	+	+
/	479.2622	17.50	1	+	+	+	+
/	479.7979	12.78	2	+	+	+	+
/	480.2565	21.76	1	+	+	+	+
/	484.2314	6.30	2	+	+	+	+
/	486.3042	14.60	1	+	+	+	+
/	486.3052	14.62	1	+	+	+	+
/	490.2600	5.59		+	+	+	+
/	493.2424	19.19	1	+	+	+	+
/	498.7538	9.63	2	+	+	+	+
/	499.2625	7.80	1	+	+	+	+
**2**	490.5942	14.40	3	-	-	-	+
/	499.7645	21.01	2	+	+	+	+
/	501.2429	6.30	1	+	+	+	+
/	501.7630	24.02	2	+	+	+	+
/	507.7421	19.80	2	+	+	+	+
/	508.2906	15.50	1	+	+	+	+
/	509.7630	19.19	2	+	+	+	+
/	510.2391	16.77	2	+	+	+	+
/	511.2845	23.66	2	+	+	+	+
/	511.7312	19.81	2	+	+	+	+
/	512.2539	16.68	2	+	+	+	+
/	512.5736	19.63	3	+	+	+	+

/	512.7552	24.02	2	✚	✚	✚	✚
/	513.7482	20.49	2	✚	✚	✚	✚
/	514.2510	13.59	2	✚	✚	-	✚
/	515.2510	19.50	2	✚	✚	✚	✚
/	515.3429	10.06	1	✚	✚	✚	✚
/	517.6038	16.04	3	✚	✚	-	✚
/	518.7344	19.81	2	✚	✚	✚	✚
/	520.7371	24.02	2	✚	✚	✚	✚
/	525.7364	25.65	2	-	-	✚	✚
/	526.2750	5.31	1	✚	✚	✚	✚
/	526.7177	19.81	2	✚	✚	✚	✚
/	527.2957	12.10	1	✚	✚	✚	✚
/	529.6020	19.40	3	✚	✚	✚	✚
/	530.7887	18.89	2	✚	✚	✚	✚
/	530.9570	19.63	3	✚	✚	✚	✚
/	531.2907	12.27	1	✚	✚	✚	✚
/	531.7855	12.78	2	✚	✚	✚	✚
/	534.1999	19.81	2	✚	✚	✚	✚
/	534.2432	6.30	1	✚	✚	✚	✚
/	535.2696	14.08	1	✚	✚	✚	✚
/	535.8036	17.75	2	✚	✚	✚	✚
/	538.2274	12.65	2	✚	✚	✚	✚
/	538.2836	19.63	3	✚	✚	✚	✚
/	538.7866	17.27	2	✚	✚	✚	✚
/	539.7781	28.50	2	✚	✚	✚	✚
/	542.7990	14.46	2	✚	✚	✚	✚
/	543.6046	19.63	3	✚	✚	✚	✚
/	544.2973	12.40	1	✚	✚	✚	✚
/	544.3862	26.81	1	✚	✚	✚	✚
/	544.7864	16.68	2	✚	✚	✚	✚
/	544.7866	17.43	2	✚	✚	✚	✚
/	545.3081	14.46	2	✚	✚	✚	✚
/	546.7834	16.04	2	✚	✚	✚	✚
/	547.7753	27.08	2	✚	✚	✚	✚
/	548.9296	19.63	3	✚	✚	✚	✚
/	550.7959	14.24	2	✚	✚	✚	✚
/	552.6142	12.58	3	✚	✚	✚	✚
/	553.3052	13.59	2	✚	✚	✚	✚
/	553.7987	13.56	2	✚	✚	✚	✚
/	556.3212	12.55	1	✚	✚	✚	✚
/	556.7927	17.20	2	✚	✚	✚	✚
/	558.3128	5.59		✚	✚	✚	✚

/	558.7653	27.09	2	+	+	+	+
/	560.3174	13.31	1	+	+	+	+
/	561.3171	24.91	1	+	+	+	+
/	562.2794	12.78	1	+	+	+	+
/	563.8012	17.94	2	+	+	+	+
/	564.2834	15.75	1	+	+	+	+
/	564.2942	13.60	2	+	+	+	+
/	568.2868	6.30	1	+	+	+	+
/	569.6130	14.46	3	+	+	+	+
/	569.7932	10.06	2	+	+	+	+
/	570.3125	12.10	1	+	+	+	+
/	571.2687	13.00	1	-	+	+	+
/	571.2819	12.27	1	+	+	+	+
/	572.2779	13.60	2	+	+	+	+
/	572.3803	25.69	1	+	+	+	+
/	573.2504	14.46	2	+	+	+	+
/	573.2644	5.31	1	+	+	+	+
/	573.3398	23.14	1	+	+	+	+
/	574.2337	27.09	2	+	+	+	+
/	575.3018	14.46	2	+	+	+	+
/	578.2752	5.31	1	+	+	+	+
/	578.3000	12.27	2	+	+	+	+
/	578.3056	12.53	1	+	+	+	+
/	579.7647	13.59	2	+	+	+	+
/	581.2635	19.63	2	+	+	+	+
/	581.3193	11.52	1	+	+	+	+
/	581.8293	17.50	2	+	+	+	+
/	583.6149	14.46	3	+	+	-	+
/	583.8135	12.10	2	+	+	+	+
/	584.3129	6.30		+	+	+	+
/	586.3359	16.42	1	+	+	+	+
/	587.1737	14.08	1	+	+	+	+
/	588.8368	16.04	2	+	+	+	+
/	589.2899	12.40	3	+	+	-	+
/	589.3338	15.44	1	+	+	+	+
/	594.2725	14.46	2	+	+	+	+
/	596.3298	12.10	1	+	+	+	+
/	596.8323	14.97	2	+	+	+	+
/	599.3923	22.27	1	+	+	+	+
/	600.2304	12.78		+	+	+	+
/	600.3867	20.13	1	+	+	+	+
/	601.3122	18.39	2	+	+	+	+

/	602.2936	14.24	1	✚	✚	✚	✚
/	602.3176	17.10	3	✚	✚	-	✚
/	602.3390	8.27	1	✚	✚	✚	✚
/	603.2689	22.63	2	✚	✚	✚	✚
/	604.2905	12.35	1	✚	✚	✚	✚
/	604.3075	9.40	1	✚	✚	✚	✚
/	607.2546	15.50	1	✚	✚	✚	✚
/	607.2905	19.63	1	✚	✚	✚	✚
/	607.8255	14.80	2	✚	✚	✚	✚
/	608.3549	16.74	1	✚	✚	✚	✚
/	609.8244	17.75	2	✚	✚	✚	✚
/	618.3392	12.35	1	✚	✚	✚	✚
/	621.3747	21.95	1	✚	✚	✚	✚
/	624.3507	15.75	1	✚	✚	✚	✚
/	628.3564	12.10	2	✚	✚	✚	✚
/	629.8132	12.78	2	✚	✚	✚	✚
/	641.3310	23.36	2	✚	✚	✚	✚
/	643.3687	16.42	1	✚	✚	✚	✚
/	644.3510	10.06	1	✚	✚	✚	✚
/	646.7862	21.95	2	✚	✚	✚	✚
/	647.3307	17.75	1	✚	✚	✚	✚
/	648.3511	20.10	2	✚	✚	✚	✚
/	648.8497	23.36	2	✚	✚	✚	✚
/	649.3000	19.82	1	✚	✚	✚	✚
/	649.3232	22.63	2	✚	✚	✚	✚
/	654.3328	17.94	2	✚	✚	✚	✚
/	655.3567	15.28	1	✚	✚	-	✚
/	655.3930	13.03	1	✚	✚	✚	✚
/	656.8466	21.95	2	✚	✚	✚	✚
/	660.3497	15.00	2	✚	✚	✚	✚
/	662.3516	17.34	2	✚	✚	✚	✚
/	664.8443	20.70	2	✚	✚	✚	✚
/	666.8469	13.20	2	✚	✚	✚	✚
/	670.3497	15.50	2	✚	✚	✚	✚
/	670.4318	23.36	1	✚	✚	✚	✚
/	672.3542	18.90	2	✚	✚	✚	✚
/	672.8430	20.00	2	✚	✚	✚	✚
/	677.3781	10.98	1	✚	✚	✚	✚
/	683.8020	20.49	2	✚	✚	✚	✚
/	688.4038	20.13	1	✚	✚	✚	✚
**3**	693.8432	26.70	2	-	-	-	✚
/	694.4061	11.52	1	✚	✚	✚	✚

/	703.3402	14.46	2	+	+	+	+
/	708.3562	18.89	2	+	+	+	+
/	708.6879	14.08	3	+	+	+	+
/	710.8729	15.35	2	+	+	+	+
**4**	713.6902	17.79	3	-	-	-	+
/	718.2710	14.08	1	+	+	+	+
/	723.3107	13.49	1	+	+	+	+
/	724.3671	13.88	2	+	+	-	+
/	726.3114	20.13	2	+	+	+	+
/	726.3605	11.86	1	+	+	+	+
/	730.3692	16.42	2	+	+	+	+
/	731.8613	12.90	2	+	+	-	+
/	732.3417	17.04	1	+	+	+	+
/	745.3828	5.59		+	+	+	+
/	745.8657	15.95	2	+	+	-	+
/	748.3914	12.40	1	+	+	+	+
/	749.0318	14.89	3	+	+	+	+
/	753.8639	14.89	2	+	+	+	+
/	755.7079	15.33	3	+	+	+	+
/	761.0394	14.46	3	+	+	+	+
/	762.3766	22.94	3	-	+	-	+
/	762.8742	13.03	2	+	+	-	+
/	766.7067	13.88	3	+	+	+	+
/	771.3333	10.39	1	+	+	+	+
/	773.3523	12.10		+	+	+	+
**5**	773.9237	18.51	2	-	-	-	+
**6**	774.9121	17.79	2	-	-	-	+
/	775.9012	16.04	2	+	+	-	+
/	778.3712	14.88	2	+	+	+	+
/	780.9306	20.10	2	-	+	+	+
/	781.9127	22.43	2	+	+	+	+
/	781.9374	21.52	2	+	+	+	+
/	783.4554	13.03	1	+	+	+	+
/	784.8969	20.13	2	+	+	+	+
/	785.8968	19.81	2	+	+	+	+
/	792.8801	18.34	2	+	+	+	+
/	793.8986	19.40	2	+	+	+	+
/	795.9306	19.61	2	+	+	+	+
/	798.9020	16.04	2	+	+	-	+
/	804.3942	18.11	2	+	+	-	+
/	804.8810	13.59	2	+	+	+	+
/	811.4610	12.30	1	+	+	+	+

/	811.4623	12.27	1	✚	✚	✚	✚
/	811.4634	12.10	1	✚	✚	✚	✚
/	811.7179	18.34	3	✚	✚	✚	✚
/	813.4302	15.75	1	✚	✚	✚	✚
/	816.4244	16.24	2	✚	✚	✚	✚
/	821.3904	21.20	1	✚	✚	✚	✚
/	824.4354	19.63	2	✚	✚	✚	✚
/	834.7436	17.43	3	✚	✚	✚	✚
/	841.4766	20.13	1	✚	✚	✚	✚
/	845.4240	22.27	3	✚	✚	✚	✚
/	845.9125	15.33	2	✚	✚	✚	✚
/	849.7429	26.80	3	-	✚	-	✚
/	850.4249	21.02	3	✚	✚	✚	✚
/	852.4311	17.04	1	✚	✚	✚	✚
/	854.4121	14.46	2	✚	✚	✚	✚
/	868.4834	10.62	1	✚	✚	✚	✚
/	874.5250	31.78	1	✚	✚	✚	✚
/	874.7801	17.92	3	✚	✚	✚	✚
/	874.9170	14.46	2	✚	✚	-	✚
/	883.4320	12.40	2	✚	✚	-	✚
/	892.5215	14.62	1	✚	✚	✚	✚
/	898.5327	16.42	1	✚	✚	✚	✚
/	926.5504	23.66	1	✚	✚	✚	✚
/	928.4948	12.27	1	✚	✚	✚	✚
/	934.4512	17.75	1	✚	✚	✚	✚
/	1014.4776	19.81	1	✚	✚	✚	✚
/	1018.5114	19.19	1	✚	✚	✚	✚
/	1021.5555	23.66		✚	✚	✚	✚
/	1027.4908	13.59	1	✚	✚	-	✚
/	1029.4871	19.50	1	✚	✚	✚	✚
/	1088.5613	17.34	1	✚	✚	✚	✚
/	1092.5548	16.04	1	✚	✚	✚	✚
/	1094.5447	27.08	1	✚	✚	✚	✚
/	1105.5997	13.59	1	✚	✚	✚	✚
/	1149.5944	14.46	1	✚	✚	✚	✚
/	1192.6607	15.00	1	✚	✚	✚	✚

Notes: Candidates were firstly screened from BPC of standard substance of pig-hide gelatin. Subsequently, they further confirmed by comparing EIC of four animal species. "✚" indicates that the marker candidate was detected by UPLC-Q-TOF-MS. "-" indicates that the marker candidate was not detected. These marker candidates showed two/three/four charges and were deduced to be peptides.
